# Early divergence of treatment response trajectories to adalimumab or its biosimilar in active ankylosing spondylitis: consensus clustering analysis of a randomized controlled trial

**DOI:** 10.3389/fimmu.2026.1804751

**Published:** 2026-06-23

**Authors:** Zhiqiang Zhong, Hongjuan Lu, Ting Li, Lingying Ye, Ling Zhou, Xiaobing Wang, Xin Wu, Huji Xu

**Affiliations:** 1 Department of Rheumatology and Immunology, Shanghai Changzheng Hospital, Naval Medical University, Shanghai, China; 2 National Key Laboratory for Immunity and Inflammation, Shanghai, China; 3 Peking-Tsinghua Center for Life Sciences, Tsinghua University, Beijing, China

**Keywords:** adalimumab, ankylosing spondylitis, biosimilar, cluster, heterogeneity, tumor necrosis factor inhibitors

## Abstract

**Background:**

Patients with active ankylosing spondylitis (AS) exhibit substantial heterogeneity in their clinical responses to tumor necrosis factor alpha inhibitors (TNFi). Consensus clustering, an unsupervised cluster discovery method, may identify AS subgroups with more homogeneous treatment response patterns to adalimumab (ADA), a widely prescribed TNFi.

**Methods:**

We performed longitudinal consensus clustering based on 8 repeated measurements of 10 core response variables in 438 patients with active AS enrolled in a 24-week phase III randomized controlled trial of ADA or its biosimilar, IBI303. Baseline characteristics and important endpoints—including the Assessment of SpondyloArthritis International Society (ASAS)-based and Ankylosing Spondylitis Disease Activity Score Inactive Disease (ASDAS)-based response criteria—were compared between the identified clusters. Predictive models of cluster membership reconstructed from data beyond week 2 were developed and internally validated to facilitate early, prospective identification of the clusters based on baseline and week-2 data.

**Results:**

Two longitudinal clusters were characterized: a favorable-response cluster (C1, n = 246, 56.2%) and a less favorable-response cluster (C2, n = 192, 43.8%). Compared with C1, C2 was characterized by older age, longer disease duration, and more frequent prior TNFi exposure, despite comparable baseline C-reactive protein (CRP) and erythrocyte sedimentation rate (ESR). For the majority of clinical endpoints except CRP and BASMI, significant divergence emerged as early as week 2 and was sustained through week 24. For instance, C1 achieved significantly higher rates of ASDAS-Inactive Disease (ASDAS-ID) than C2 at week 2 (0.21 [95% CI 0.16, 0.26] vs. 0.01 [95% CI 0.00, 0.02]; Risk Difference [RD]: -0.21 [95% CI -0.26, -0.15], p < 0.001) and week 24 (0.63 [95% CI 0.57, 0.69] vs. 0.14 [95% CI 0.09, 0.18]; RD: -0.49 [95% CI -0.57, -0.42], p < 0.001). A multivariable model incorporating one baseline and eight week-2 variables had an optimism-corrected C-statistic of 0.880 [95% CI 0.859, 0.915] to correctly identify C2.

**Conclusions:**

These observed response trajectories exhibited distinct baseline characteristics and early response divergence at week 2. While these findings provide a hypothesis-generating framework for early patient stratification, their utility for prospective clinical patient triage requires further independent validation.

## 1 Introduction

Ankylosing spondylitis (AS), a prototype of axial spondyloarthritis (axSpA), the umbrella term describing chronic inflammation of the spinal and sacroiliac joints with etiologies not fully elucidated (1), is highly heterogeneous in terms of disease manifestation and responses to pharmacotherapy (2). Over the last two decades, novel cytokine- or pathway-targeted therapies have been developed, profoundly expanding the therapeutic armamentarium, among which tumor necrosis factor alpha inhibitors (TNFi) have been the most widely prescribed agents worldwide (3, 4). While it is well established that TNFi are superior on average to placebo in alleviating active axial symptoms in patients with AS, as robustly demonstrated in randomized controlled trials (RCTs) (5), more than one-third of patients did not respond adequately at 3 months (6), as defined by improvement of the Ankylosing Spondylitis Disease Activity Score (ASDAS) of less than 1.1 points from baseline (7). It is therefore of great importance to stratify the population into more homogeneous subgroups in terms of response to therapy, also known as theratypes (8), ideally before treatment initiation or earlier in the course of TNFi therapy, so as to minimize unnecessary drug exposure and facilitate more efficient drug development (9). A first reasonable step toward this goal is to explore the presence of inherent clusters based on the longitudinal trajectories of pertinent endpoints.

The primary endpoints used in AS RCTs comprise multifaceted questionnaires and laboratory data (10), yielding mixed-type information that may not be fully exploited by conventional analysis. Retrospective comparisons between “responders” and “non-responders” rely on response criteria with thresholds that often vary among different populations (11). Controversies exist regarding which endpoint and cut-off value to choose (12). More importantly, analyzing binary endpoints at fixed time points discards the granularity and dynamics of longitudinal data, leading to potential information loss. In contrast, unsupervised clustering methods can make full use of longitudinal data without being constrained by predefined thresholds. In this study, we applied consensus clustering, a resampling-based approach (13) that simultaneously accounts for multivariate longitudinal mixed-type data to uncover subtypes with more homogeneous short-term responses to TNFi in patients with active AS. To capture the full picture of disease course, repeated measures of all non-redundant components of the ASDAS, Assessment of SpondyloArthritis International Society (ASAS) (14), and Bath Ankylosing Spondylitis Disease Activity Index (BASDAI) (15) response criteria were chosen as inputs to the consensus clustering algorithm.

To the best of our knowledge, clustering analyses of treatment response in AS are scarce. Previous studies have solely examined ASDAS trajectories in axSpA, either in response to etanercept (16) or regardless of TNFi exposure (17, 18). Differentiation in ASDAS as early as 6 months was observed and appeared to be maintained through 2 years (16), supporting the predictive value of short-term responses as reported in previous analyses (19, 20). Due to the retrospective nature of these studies, high-resolution data are still lacking, precluding earlier identification of response subtypes. The present study aimed to address the clinical heterogeneity of treatment response by taking advantage of a completed RCT, with prospectively, regularly, and intensively collected follow-up data.

## 2 Materials and methods

### 2.1 Study design and study population

This *post-hoc* analysis was based on de-identified individual participant data from a phase III multicenter randomized controlled trial that established the equivalence of the originator adalimumab (ADA) and its biosimilar IBI303 in Chinese patients with active AS (21). The original study was approved by the ethics committees of Shanghai Changzheng Hospital (approval no 2016-17). The study design and eligibility criteria have been detailed elsewhere. Briefly, 438 patients who fulfilled the 1984 Modified New York criteria for AS (22) and had active disease despite a 4-week course of non-steroidal anti-inflammatory drugs (NSAIDs) therapy, were randomized 1:1 to receive subcutaneous adalimumab or IBI303–40 mg every other week until week 22. Outcomes were measured at baseline, week 2, week 4, and every 4 weeks thereafter until week 24.

### 2.2 Consensus clustering

To facilitate unsupervised cluster discovery, we employed consensus clustering to characterize the multivariable signatures of treatment response. A total of 10 disease activity-related variables, recorded at all eight visits, were selected as the basis for consensus clustering. These variables encompass the core components of the widely used outcome measures in AS trials, i.e., the ASDAS, ASAS, and BASDAI scores (**Table 1**).

**Table 1:** Variables selected as the basis for the consensus clustering analysis of disease activity.

Variable	Scale	Component(s) of
Fatigue	NRS 0-10	BASDAI Q1
Back pain (total + nocturnal average)	NRS 0-10	BASDAI Q2, ASDAS D1, ASAS D2
Peripheral pain	NRS 0-10	BASDAI Q3, ASDAS D4
Enthesitis	NRS 0-10	BASDAI Q4
Morning stiffness severity	NRS 0-10	BASDAI Q5, ASAS D4
Morning stiffness duration	NRS 0-10	BASDAI Q6, ASDAS D2, ASAS D4
Patient Global Assessment (PtGA)	NRS 0-10	ASAS D1, ASDAS D3
CRP	mg/L	ASDAS D5, ASAS D5
BASFI	NRS 0-10	ASAS D3
BASMI	NRS 0-10	ASAS D6

NRS, Numerical Rating Scale; BASDAI, Bath Ankylosing Spondylitis Disease Activity Index; ASDAS, Ankylosing Spondylitis Disease Activity Score; ASAS, Assessment of SpondyloArthritis International Society; CRP, C-reactive protein; BASFI, Bath Ankylosing Spondylitis Functional Index; BASMI, Bath Ankylosing Spondylitis Metrology Index. Q1–Q6, Question 1–6; D1–D6, Domain 1–6.

The trajectories of all 10 variables were fitted with a mixture of generalized additive models (GAMs) using regression splines of 4 degrees of freedom to flexibly capture the non-linear time course of the outcomes. We also tested different choices of model forms, degrees of freedom, and response outcomes, with highly consistent clustering results (**Supplementary Figures 7**, **9**).

Consensus clustering assignment was obtained by iterative model fitting, exploring 50 bootstrapped subsamples (80% of the total sample), and cluster solutions of 2 to 5 were evaluated using hierarchical clustering with Ward’s minimum variance linkage. The optimal number of clusters was determined following published guidance (13, 23, 24), specifically by inspecting the consensus matrix (clean separation of diagonal blocks), consensus cumulative distribution function (CDF) plot (flat middle segment), and density distribution of the consensus index (values concentrated near 0 and 1). To quantitatively measure how well the different cluster solutions separated participants, the following complementary metrics were examined: the proportion of ambiguous clustering (PAC, which quantifies the flatness of the CDF middle segment and the ambiguity of clustering; the lower, the better; 0 indicates perfect separation), the delta area plot (the cluster number that maximizes the delta values), item consensus (consistency of values for each item), and cluster-consensus statistics (for which higher consensus index values indicate a higher degree of consensus).

### 2.3 Cluster comparisons

The prognostic implications of the identified clusters were evaluated by comparing the following pertinent outcomes, including changes from baseline (CfB) in ASDAS-C-reactive protein (CRP), BASDAI, BASFI, BASMI, CRP, patient global assessment (PtGA), and proportions of patients achieving ASAS20, ASAS40, ASAS-PR, ASAS5/6, ASDAS-CII, ASDAS-MI, and ASDAS-ID at each follow-up visit until week 24. The definition of these outcomes can be found elsewhere (7, 14).

For continuous endpoints, between-group differences in CfB data at each follow-up visit were estimated with mixed models for repeated measures (MMRM) under the assumption of missing at random (MAR). For binary outcomes, generalized estimating equations (GEE) were fitted. To comply with the Consolidated Standards of Reporting Trials (CONSORT) recommendations for reporting both relative and absolute effects (25), odds ratios (ORs) and risk differences (RDs) were estimated using the logit-link and identity-link functions, respectively (26). For both MMRM and GEE models, cluster assignment, follow-up visit, and their interaction were included as fixed effects, and body weight was included as a covariate. In addition, the baseline level of each continuous endpoint and its interaction with visit were further included in the GEE model. The covariance structure was specified as first-order autoregressive, based on preliminary analyses indicating superior model convergence compared with an unstructured covariance structure. Data missingness was addressed by MMRM (27) and non-responder imputation (NRI) (28) for continuous and binary endpoints, respectively. Results of the complete case analysis were also presented. In addition, sensitivity analysis with the δ-adjustment approach within the pattern mixture modeling (PMM) framework was also performed (29) (details are provided in Section 2 of the **Supplementary Material**).

### 2.4 Predictive modeling of the observed clusters

To predict the identified clusters, we further developed multivariable logistic regression models using baseline data (Model 1) and baseline plus week-2 data (Model 2). To prevent predictor-outcome overlap, clusters were reconstructed using post-week 2 data (95.6% concordance with original clustering; see **Supplementary Figure 12**). LASSO-guided model training and internal validation were conducted using robust bootstrap resampling methods (30, 31). Optimism-corrected performance metrics, including discrimination (C-statistic or area under the curve (AUC)), calibration (slope, intercept, and integrated calibration index (ICI)), and overall accuracy (Brier score) were reported. Models were compared using continuous net reclassification improvement (NRI) and integrated discrimination improvement (IDI) (32). Detailed methods are provided in the **Supplementary Material**. Results of the first imputed dataset are presented, with other datasets checked for consistency.

### 2.5 Other considerations

Descriptive data were not imputed and are presented as mean ± SD, median (interquartile range), and number (percentage) for normally distributed continuous variables, non-normally distributed continuous variables, and categorical variables, respectively. All analyses were performed with R statistical software (33), with the longmixr (13) package for consensus clustering, the MMRM (34) and geepack (35) packages for modeling longitudinal repeated measures, and the emmeans (36) package for extracting marginal means and pairwise comparisons from fitted models. Detailed analysis procedures are provided in the **Supplementary Material**.

## 3 Results

### 3.1 Results of consensus clustering

**Figure 1:**
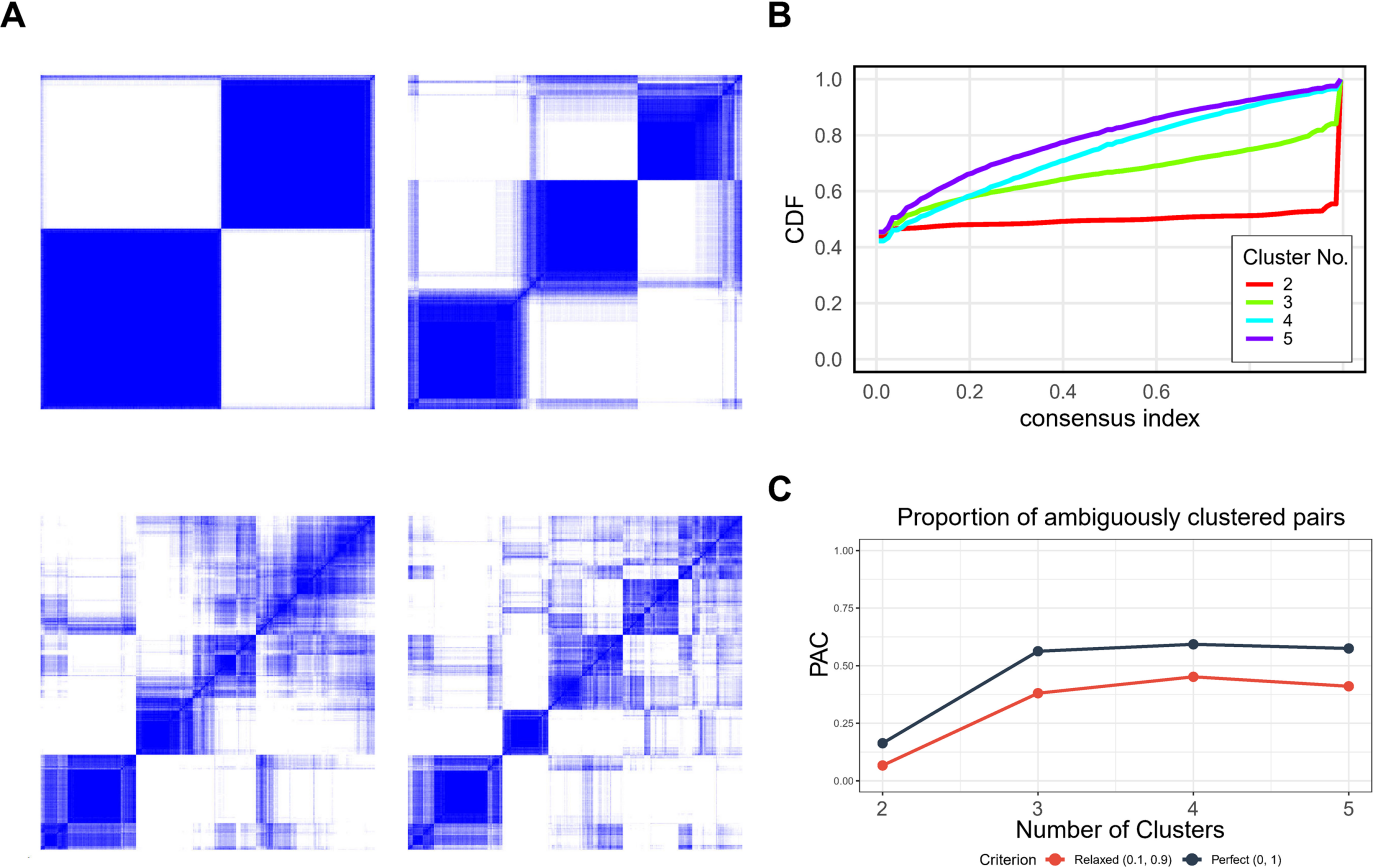
Diagnostic plots for determining the optimal number of clusters. Consensus matrix plot **(A)**, consensus cumulative distribution function **(B)**, and proportion of ambiguously clustered pairs **(C)** to determine the optimal number of clusters of different clustering with cluster numbers ranging from 2 to 5. Perfect criteria correspond to an ambiguous range of the open interval (0, 1), while relaxed criteria correspond to (0.1 to 0.9).

Based on the diagnostic metrics detailed in Section 2.2, we evaluated the clustering solutions to explore the heterogeneity of treatment responses. The consensus matrix revealed a 2-block diagonal structure with relatively distinct boundaries (**Figure 1A**), suggesting that the cohort predominantly segregates into two main clusters. The 2-cluster solution also produced a CDF curve characterized by a flatter middle segment and an abrupt increase approaching 1 (**Figure 1B**). Intuitively, this specific shape indicates that the cluster assignments are highly decisive: patient pairs are either rarely grouped together (consensus index near 0) or almost always grouped together (consensus index near 1). Ambiguous intermediate consensus index values are rare, as reflected by the horizontal middle segment (13, 23). Cluster ambiguity, as measured by PAC, also supports the 2-cluster solution, as K = 2 yielded the lowest PAC (**Figure 1C**), indicating the least ambiguous clustering (24). Other complementary diagnostic plots consistently reaffirmed K = 2 as the least unstable clustering solution (including the histogram of consensus index, delta area, item-consensus, and cluster-consensus plots; **Supplementary Figure 6**).

Consequently, we characterized two clusters, hereafter designated as C1 (Consensus Cluster 1, n = 246, 56.2%) and C2 (Consensus Cluster 2, n = 192, 43.8%), exhibiting differential responses to adalimumab and its biosimilar IBI303 (**Supplementary Figure 10**). A cluster solution of 4 was also examined; it manifested as subdivisions of the 2-cluster solution by baseline disease scores (**Supplementary Figure 11**).

### 3.2 Baseline characteristics

The baseline characteristics of C1 and C2 are presented in **Table 2**. The two clusters differed significantly in many aspects, including demographics, HLA-B27 positivity, disease stage, baseline disease activity, and previous exposure to TNFi. Patients in the C2 cluster (less favorable responders) were older (34.0 [28.0, 39.5] years vs. 30.0 [26.0, 36.0] years), shorter (median 170.0 [165.0, 174.5] cm vs. 171.0 [167.0, 175.0] cm), had a lower prevalence of HLA-B27 (86.5% vs. 94.7%), longer symptom duration (10.0 [6.0, 14.0] years vs. 8.0 [5.0, 11.0] years), and a higher rate of prior TNFi exposure (49.0% vs. 36.6%) (all p < 0.05). Baseline disease activity or functional impairment were more severe in C2, including higher Total Back Pain scores (7.6 [6.8, 8.6] vs. 6.6 [5.7, 7.7]) and BASFI (6.0 [4.7, 7.1] vs. 4.0 [2.2, 5.6]) (both p < 0.001). In contrast, objective indicators of inflammation were comparable between the two clusters (C-reactive protein: 9.2 [3.1, 22.9] mg/L vs. 10.1 [3.9, 22.6] mg/L, p = 0.509; erythrocyte sedimentation rate: 20.0 [9.0, 40.0] mm/h vs. 17.0 [9.0, 34.0] mm/h, p = 0.381).

**Table 2:** Baseline characteristics of patients in the C1 and C2 clusters.

Characteristics	C1 (N = 246)	C2 (N = 192)	p
Demographics			
Male	205 (83.3%)	157 (81.8%)	0.763
Age	30.0 (26.0, 36.0)	34.0 (28.0, 39.5)	< 0.001
Height, cm	171.0 (167.0, 175.0)	170.0 (165.0, 174.5)	0.039
Weight, kg	66.8 (58.0, 76.0)	67.0 (58.0, 76.0)	0.750
BMI, kg/m^2^	22.8 (20.1, 25.9)	23.6 (21.1, 26.0)	0.064
Smoking history	66 (26.8%)	55 (28.6%)	0.753
Diagnosis-related			
Disease duration	4.0 (1.0, 8.0)	5.0 (2.0, 10.0)	0.003
Symptom duration	8.0 (5.0, 11.0)	10.0 (6.0, 14.0)	0.001
HLA-B27(+)	233 (94.27%)	166 (86.5%)	0.004
ASAS-related			
PtGA	6.5 (5.3, 7.4)	7.4 (6.3, 8.2)	< 0.001
Total back pain	6.6 (5.7, 7.7)	7.6 (6.8, 8.6)	< 0.001
BASFI	4.0 (2.2, 5.5)	6.0 (4.7, 7.1)	< 0.001
BASMI	3.6 (2.5, 4.6)	4.3 (3.0, 5.5)	< 0.001
BASDAI-related			
Fatigue	6.2 (5.2, 7.2)	7.3 (6.4, 8.4)	< 0.001
Back pain	6.4 (5.4, 7.6)	7.3 (6.3, 8.3)	< 0.001
Peripheral pain	5.2 (3.2, 6.6)	6.5 (5.2, 7.9)	< 0.001
Enthesitis	5.9 (4.7, 7.1)	6.9 (6.0, 8.0)	< 0.001
MASES	1.0 (0.0, 3.0)	2.0 (1.0, 4.5)	0.002
Inflammation biomarkers			
CRP, mg/L	10.1 (3.9, 22.6)	9.2 (3.1, 22.9)	0.509
ESR, mm/h	17.0 (9.0, 34.0)	20.0 (9.0, 40.0)	0.381
Previous therapy			
MTX	65 (26.4%)	63 (32.8%)	0.176
SASP	161 (65.4%)	117 (60.9%)	0.383
Steroids	27 (11.0%)	23 (12.0%)	0.860
TNFi	90 (36.6%)	94 (49.0%)	0.012

BMI, body mass index; HLA-B27, human leukocyte antigen B27; PtGA, Patient Global Assessment; BASFI, Bath Ankylosing Spondylitis Functional Index; BASMI, Bath Ankylosing Spondylitis Metrology Index; MASES, Maastricht Ankylosing Spondylitis Enthesitis Score; CRP, C-reactive protein; ESR, erythrocyte sedimentation rate; MTX, methotrexate; SASP, sulfasalazine; TNFi, tumor necrosis factor inhibitor.

### 3.3 Disease outcomes across C1 and C2 clusters

#### 3.3.1 Continuous endpoints

**Figure 2:**
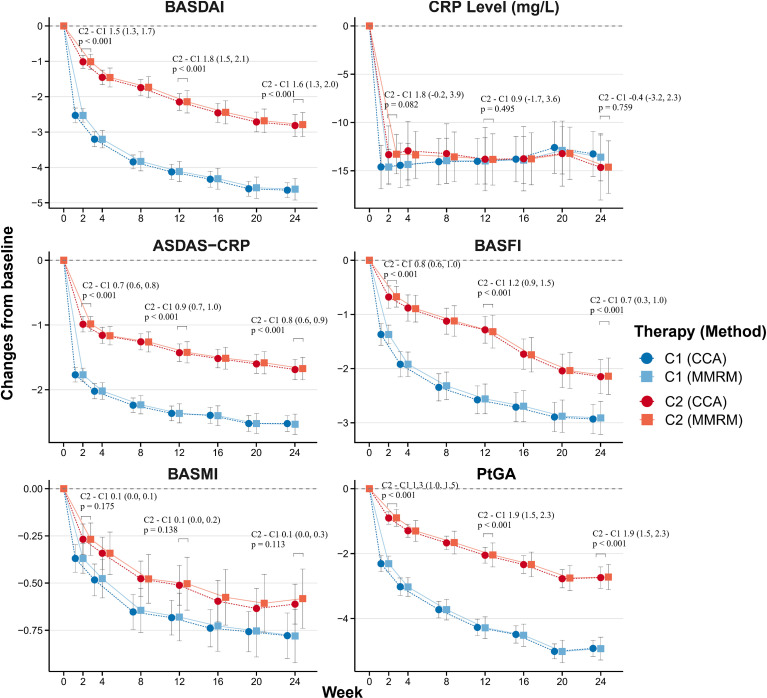
Changes from baseline of continuous endpoints through week 24 in the C1 cluster (blue) and C2 cluster (red). Results from both mixed models for repeated measures (MMRM) (circles and solid lines) and complete case analysis (CCA, rectangles and dashed lines) are presented.

The dynamic changes of seven key continuous endpoints over 24 weeks in each cluster are depicted in **Figure 2**, and the between-group differences at weeks 2, 12, and 24 are summarized in **Table 3**. Results of the MMRM and complete case analysis are presented and overlapped closely.

**Table 3:** Changes from baseline of numeric outcomes in the C1 and C2 clusters and their differences at weeks 2, 12, and 24.

Changes from baseline	C11 (N = 246)	C21 (N = 192)	Difference between C2 and C11	*p* ^2^
ASDAS-CRP				
Week 2	-1.7 (-1.8, -1.6)	-1.0 (-1.1, -0.9)	0.7 (0.6, 0.8)	< 0.001
Week 12	-2.3 (-2.5, -2.2)	-1.5 (-1.6, -1.3)	0.9 (0.7, 1.0)	< 0.001
Week 24	-2.5 (-2.6, -2.3)	-1.7 (-1.9, -1.5)	0.8 (0.6, 0.9)	< 0.001
BASDAI				
Week 2	-2.5 (-2.7, -2.3)	-1.0 (-1.2, -0.8)	1.5 (1.3, 1.7)	< 0.001
Week 12	-4.0 (-4.3, -3.7)	-2.2 (-2.5, -1.9)	1.8 (1.5, 2.1)	< 0.001
Week 24	-4.5 (-4.8, -4.2)	-2.9 (-3.2, -2.5)	1.6 (1.3, 2.0)	< 0.001
BASFI				
Week 2	-1.4 (-1.6, -1.2)	-0.6 (-0.8, -0.4)	0.8 (0.6, 1.0)	< 0.001
Week 12	-2.5 (-2.8, -2.2)	-1.4 (-1.7, -1.0)	1.2 (0.9, 1.5)	< 0.001
Week 24	-2.9 (-3.2, -2.6)	-2.2 (-2.5, -1.8)	0.7 (0.3, 1.0)	< 0.001
BASMI				
Week 2	-0.4 (-0.4, -0.3)	-0.3 (-0.4, -0.2)	0.1 (-0.0, 0.1)	0.175
Week 12	-0.6 (-0.8, -0.5)	-0.5 (-0.7, -0.4)	0.1 (0.0, 0.2)	0.138
Week 24	-0.7 (-0.9, -0.6)	-0.6 (-0.8, -0.5)	0.1 (0.0, 0.3)	0.113
CRP, mg/L				
Week 2	-14.8 (-16.6, -13.0)	-13.0 (-15.1, -10.9)	1.8 (-0.2, 3.9)	0.082
Week 12	-14.3 (-16.7, -12.0)	-13.4 (-16.1, -10.7)	0.9 (-1.7, 3.6)	0.495
Week 24	-13.9 (-16.3, -11.4)	-14.3 (-17.1, -11.5)	-0.4 (-3.2, 2.3)	0.759
PtGA				
Week 2	-2.2 (-2.5, -2.0)	-1.0 (-1.2, -0.7)	1.3 (1.0, 1.5)	< 0.001
Week 12	-4.1 (-4.4, -3.8)	-2.2 (-2.6, -1.8)	1.9 (1.5, 2.3)	< 0.001
Week 24	-4.8 (-5.1, -4.4)	-2.9 (-3.3, -2.5)	1.9 (1.5, 2.3)	< 0.001

1 Estimate (95% confidence interval).

2 After Bonferroni multiple comparison correction for multiple pairwise time points comparisons within each outcome measure.

While patients in both clusters experienced statistically significant improvements from baseline in all these endpoints at weeks 2, 12, and 24, the magnitudes differed between C1 and C2 clusters for several parameters. For example, the marginal estimated changes from baseline (CfB) in ASDAS-CRP were greater in C1 than in C2, yielding marginal between-group differences of 0.7 (95% CI 0.6, 0.8) at week 2, 0.9 (95% CI 0.7, 1.0) at week 12, and 0.8 (95% CI 0.6, 0.9) at week 24 (all p < 0.001). In contrast, the magnitudes of improvements did not differ significantly between C1 and C2 for objective measures, including BASMI and CRP. For instance, the marginal between-group differences for CRP changes were 1.8 (95% CI -0.2, 3.9) at week 2, 0.9 (95% CI -1.7, 2.8) at week 12, and -0.4 (95% CI -3.2, 2.3) at week 24 (all p > 0.5).

#### 3.3.2 Binary endpoints

**Figure 3:**
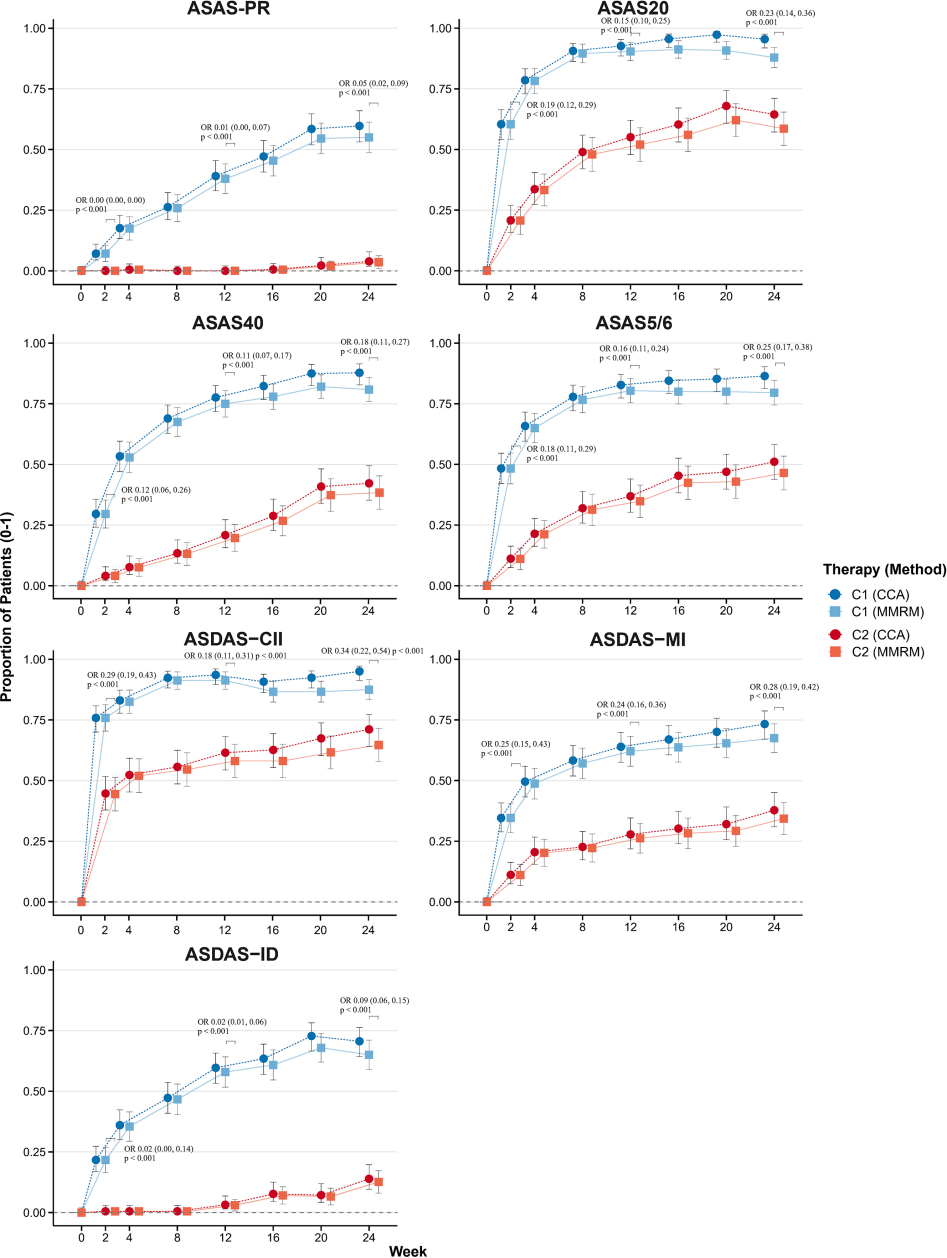
Proportions of patients in the C1 cluster (red) and C2 cluster (blue) who achieved key response criteria, including ASAS-PR, ASAS20, ASAS40, ASAS5/6, ASDAS-CII, ASDAS-MI, and ASDAS-ID. Results from both non-responder imputation (NRI) (circles and solid lines) and complete case analysis (CCA, rectangles and dashed lines) are presented.

**Figure 3** illustrates the proportion of patients achieving eight widely used response criteria throughout the 24-week study period, stratified by the C clusters. NRI and complete case analysis differed only subtly, confirming that data missingness in the present study had a negligible impact on the outcomes.

As shown in the response curves (**Figure 3**), the C1 and C2 clusters demonstrated an early-onset and sustained discrepancy in each of these criteria, from as early as week 2 through the entire follow-up period to week 24. The C1 cluster achieved far more favorable outcomes than C2 across all binary endpoints at weeks 2, 12, and 24 (**Table 4**), with consistently negative risk differences (RDs) and odds ratios (ORs) less than 1 (all p < 0.001). For instance, at week 12, C2 was associated with a lower ASAS20 rate than C1 (0.54 [95% CI 0.47, 0.61] vs. 0.88 [95% CI 0.84, 0.92]), yielding an RD of -0.35 (95% CI -0.43, -0.26) and an OR of 0.15 (95% CI 0.10, 0.25) (both p < 0.001). The difference was also significant at week 2 (RD -0.38 [95% CI -0.46, -0.29], OR 0.19 [95% CI 0.12, 0.29], both p < 0.001). For more stringent response criteria such as ASDAS-ID, the difference was more pronounced: by week 12, the proportion of C2 patients achieving ASDAS-ID was 0.03 (95% CI 0.01, 0.06), compared with 0.57 (95% CI 0.50, 0.63) in C1, corresponding to an RD of -0.53 (95% CI -0.60, -0.47) and an OR of 0.02 (95% CI 0.01, 0.06).

**Table 4:** Proportion of patients in the C1 and C2 clusters who achieved key binary endpoints at weeks 2, 12, and 24, and risk differences and odds ratios associated with the C2 cluster.

Outcomes	C2^1^ (N = 246)	C2^1^ (N = 192)	Difference between C2 and C1^1^		*p* ^2^
			Risk differences	Odds ratios	
ASAS20					
Week 2	0.59 (0.53, 0.65)	0.21 (0.16, 0.27)	-0.38 (-0.46, -0.29)	0.19 (0.12, 0.29)	< 0.001
Week 12	0.88 (0.84, 0.92)	0.54 (0.47, 0.61)	-0.35 (-0.43, -0.26)	0.15 (0.10, 0.25)	< 0.001
Week 24	0.87 (0.82, 0.91)	0.59 (0.52, 0.66)	-0.27 (-0.35, -0.19)	0.23 (0.14, 0.36)	< 0.001
ASAS40					
Week 2	0.28 (0.23, 0.34)	0.05 (0.02, 0.08)	-0.24 (-0.30, -0.17)	0.12 (0.06, 0.26)	< 0.001
Week 12	0.72 (0.66, 0.78)	0.22 (0.16, 0.28)	-0.50 (-0.58, -0.42)	0.11 (0.07, 0.17)	< 0.001
Week 24	0.79 (0.74, 0.84)	0.40 (0.33, 0.47)	-0.39 (-0.48, -0.31)	0.18 (0.11, 0.27)	< 0.001
ASAS-PR					
Week 2	0.07 (0.04, 0.10)	0.00 (0.00, 0.00)	-0.07 (-0.10, -0.04)	0.00 (0.00, 0.00)	< 0.001
Week 12	0.37 (0.31, 0.43)	0.01 (0.00, 0.02)	-0.36 (-0.42, -0.30)	0.01 (0.00, 0.07)	< 0.001
Week 24	0.56 (0.49, 0.62)	0.05 (0.02, 0.09)	-0.50 (-0.57, -0.43)	0.05 (0.02, 0.09)	< 0.001
ASAS5/6					
Week 2	0.46 (0.40, 0.52)	0.13 (0.08, 0.18)	-0.33 (-0.41, -0.25)	0.18 (0.11, 0.29)	< 0.001
Week 12	0.78 (0.73, 0.83)	0.36 (0.30, 0.43)	-0.42 (-0.50, -0.33)	0.16 (0.11, 0.24)	< 0.001
Week 24	0.78 (0.73, 0.83)	0.47 (0.40, 0.54)	-0.31 (-0.39, -0.22)	0.25 (0.17, 0.38)	< 0.001
ASDAS-CII					
Week 2	0.74 (0.69, 0.80)	0.45 (0.38, 0.52)	-0.29 (-0.38, -0.20)	0.29 (0.19, 0.43)	< 0.001
Week 12	0.89 (0.85, 0.93)	0.60 (0.53, 0.67)	-0.29 (-0.37, -0.21)	0.18 (0.11, 0.31)	< 0.001
Week 24	0.85 (0.81, 0.90)	0.67 (0.60, 0.73)	-0.19 (-0.27, -0.11)	0.34 (0.22, 0.54)	< 0.001
ASDAS-MI					
Week 2	0.34 (0.28, 0.40)	0.11 (0.07, 0.16)	-0.22 (-0.30, -0.15)	0.25 (0.15, 0.43)	< 0.001
Week 12	0.61 (0.54, 0.67)	0.27 (0.21, 0.33)	-0.33 (-0.42, -0.25)	0.24 (0.16, 0.36)	< 0.001
Week 24	0.66 (0.60, 0.72)	0.35 (0.29, 0.42)	-0.30 (-0.39, -0.21)	0.28 (0.19, 0.42)	< 0.001
ASDAS-ID					
Week 2	0.21 (0.16, 0.26)	0.01 (0.00, 0.02)	-0.21 (-0.26, -0.15)	0.02 (0.00, 0.14)	< 0.001
Week 12	0.57 (0.50, 0.63)	0.03 (0.01, 0.06)	-0.53 (-0.60, -0.47)	0.02 (0.01, 0.06)	< 0.001
Week 24	0.63 (0.57, 0.69)	0.14 (0.09, 0.18)	-0.49 (-0.57, -0.42)	0.09 (0.06, 0.15)	< 0.001

1 Estimate (95% confidence interval).

2 Only p-values for odds ratios are presented (those for risk differences showed similar statistical significance and are not presented to save space), after Bonferroni multiple comparison correction for multiple pairwise time points comparisons within each outcome measure.

### 3.4 Predictive models of the C2 cluster

As detailed in Section 3 of the **Supplementary Material**, we obtained multivariable logistic regression Model 1 and Model 2. Model 2, based on nine predictors from baseline and week-2 response data (**Table 5**, **Supplementary Table 4**), outperformed Model 1 in overall accuracy and discrimination. The optimism-corrected AUC improved from 0.733 (95% CI 0.703, 0.784) in Model 1 to 0.880 (95% CI 0.859, 0.915) in Model 2, and the scaled Brier score improved from 0.144 (95% CI 0.138, 0.150) to 0.406 (95% CI 0.399, 0.413), but calibration was similar, with the calibration slope changing from 1.325 (95% CI 1.250, 1.459) to 1.362 (95% CI 1.316, 1.495). Model comparison confirmed significant reclassification gains of Model 2 compared with Model 1 (NRI: 0.980; IDI: 0.233; both p < 0.001). Full performance metrics and visualization are provided in **Supplementary Figure 15**, **Supplementary Table 3**.

**Table 5:** Coefficients of the prediction model of the C2 cluster.

Variable	β coefficient	OR (95% CI)	Wald Z	*p*
Baseline				
Prior TNFi use	0.691	1.996 (1.158–3.441)	2.49	0.013
Week-2				
Total back pain	0.189	1.208 (0.908–1.607)	1.30	0.193
Peripheral pain	0.209	1.232 (1.021–1.486)	2.18	0.029
Enthesitis	0.159	1.172 (0.918–1.495)	1.27	0.202
Stiffness severity	0.220	1.246 (1.011–1.534)	2.06	0.039
BASFI	0.126	1.134 (0.932–1.381)	1.26	0.208
BASMI	0.179	1.196 (0.987–1.449)	1.83	0.067
PtGA	0.247	1.280 (0.996–1.643)	1.93	0.053
CRP	0.126	1.135 (1.050–1.227)	3.18	0.001
Intercept				
-6.824				

**Figure 4:**
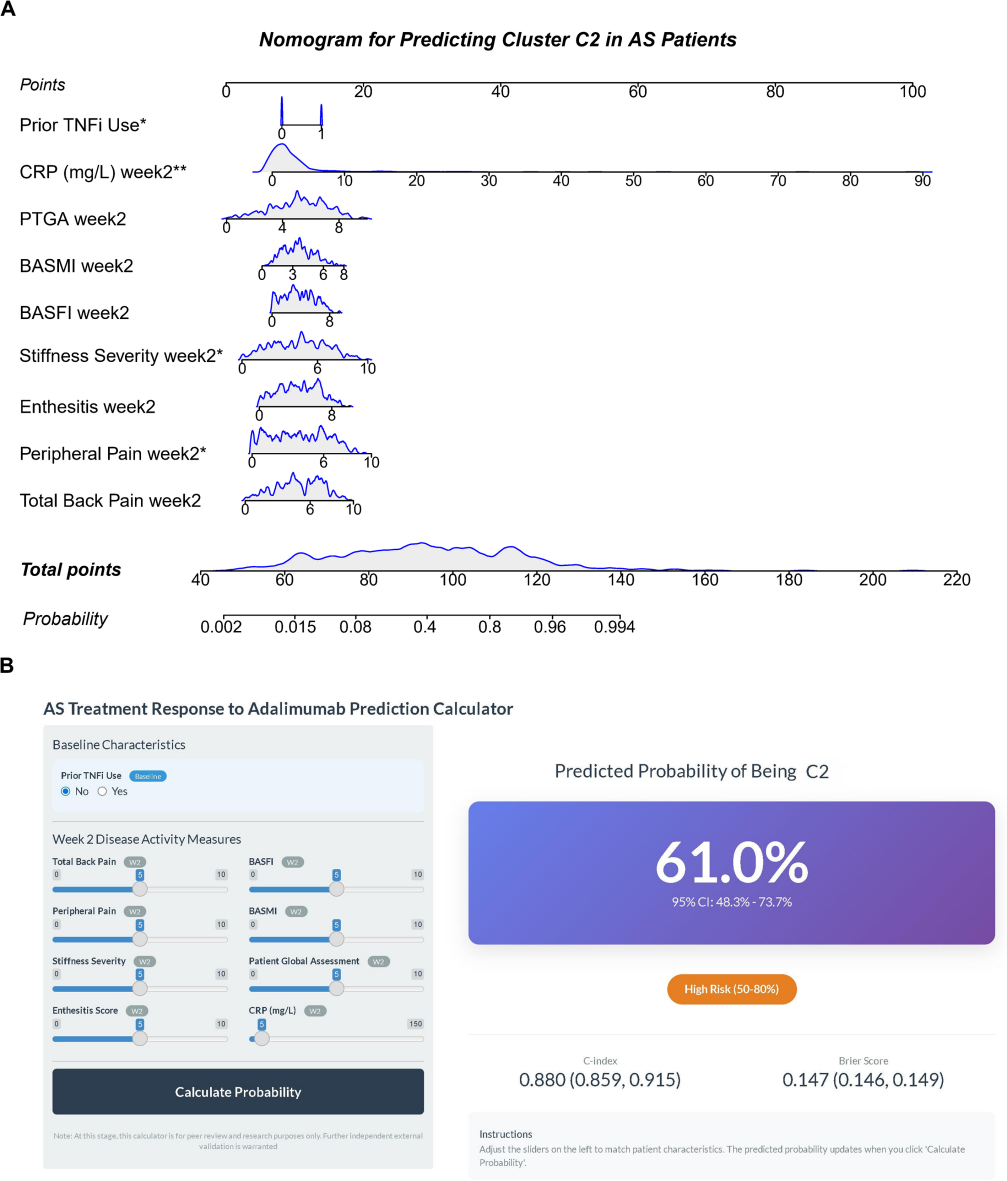
Predictive model for the C2 cluster based on baseline and week 2 data, presented as a nomogram **(A)** and an online Shiny-based calculator **(B)**.

Given its superior internal performance, Model 2 was selected for final presentation, with its coefficients, a nomogram and a Shiny-based web calculator provided to facilitate future external validation (**Table 5**, **Figure 4**).

## 4 Discussion

These results illustrate two distinct trajectories—the C1 and C2 clusters—that may represent two populations with markedly distinct baseline characteristics and divergent likelihoods of response to adalimumab or its biosimilar IBI303, as defined by a wide range of validated criteria in patients with active AS. The short-term symptom-alleviating benefits of adalimumab or IBI303 were predominantly confined to the C1 cluster. Conversely, the C2 cluster, which accounted for a substantial proportion of patients (192/438, 43.8%), experienced comparatively lower efficacy, especially with regard to more stringent response criteria such as ASAS-PR and ASDAS-ID. These findings highlight a common and longstanding theme in treating patients with rheumatologic diseases—that is, the heterogeneity of clinical phenotypes and treatment responses (37).

The absence of gold-standard diagnostic criteria for rheumatologic diseases has driven approaches to define more homogeneous patient groups, most notably through the development of classification criteria (38). Responses to treatment have also been inherent components of these criteria, as exemplified by the adoption of responsiveness to NSAIDs in the ASAS classification criteria for axial spondyloarthritis (39) and to corticosteroids in the classification criteria for polymyalgia rheumatica (40). Therefore, the newly coined concept of theratype (8), as an extension of phenotype and endotype, represents a long-standing tradition in rheumatology.

Previous trajectory studies in axSpA have provided important insights into treatment heterogeneity. For example, a small cohort of 178 axSpA patients initiating etanercept identified four ASDAS trajectories (16), while a larger, more diverse (not restricted to patients receiving TNFi) cohort (n = 708) yielded five (17, 18). These clusters broadly represent two distinct trajectories: persistently high ASDAS activity versus rapid improvement, suggesting that in varying proportions of axSpA patients, ASDAS remains stable regardless of TNFi therapy. Using 3- to 6-month assessment intervals, these studies indicated that long-term (up to 2 years) ASDAS trajectories diverged at 6 months (16, 18). For patients with AS, longer-term responses to TNFi, up to 5 years, were also correlated with short-term responses at 3 months, both in controlled trials and real-world settings (20, 41). These observations form the basis for defining TNFi non-response (42) and criteria for TNFi continuation (43).

In this *post-hoc* analysis of an AS RCT with granular follow-up data, trajectory divergence was evident as early as 2 weeks for the majority of endpoints. This is much earlier than the currently recommended 3-month timeline for TNFi efficacy assessment and triage (43), providing an opportunity for earlier identification of response trajectories. A multivariable model based on prior TNFi exposure history and early core disease measures at week 2 after adalimumab initiation can predict the less favorable C2 cluster with good internal discriminative performance (Model 2, internally validated AUC of 0.880).

Furthermore, by simultaneously examining variables of multifaceted treatment responses, ranging from subjective back pain and morning stiffness to objective inflammatory markers, our analysis provided a more comprehensive characterization of disease trajectory signatures over time. For instance, the C1 and C2 clusters experienced fewer discrepant responses in objective parameters such as CRP and BASMI than in patient-reported outcomes (PROs) such as BASFI. This subtle dissociation between objective inflammation and patient perception may be missed in conventional univariate analysis. It also suggests that the “unfavorable responder” in AS is not merely a failure of TNF blockade to suppress systemic inflammation, but may involve distinct mechanisms not targeted by TNFi, such as centralized pain sensitization (44) or pharmacogenetics (2), that may contribute disproportionately to subjective symptoms.

Such mechanistic complexity likely explains the challenges of predicting treatment responses in axSpA patients based solely on routinely collected pretreatment characteristics. Prior efforts using various machine-learning techniques have consistently reported suboptimal discriminative performance even in the training datasets, with the best AUC being 0.77 for ASDAS non-response (6) and 0.65 for ASAS20 (45).

In our study, the baseline characteristics of the unfavorable responders (C2)—including prior TNFi exposure, higher body mass index (BMI), longer disease duration, and lower HLA-B27 prevalence—align with prognostic factors identified in previous cohorts (6, 45–47). Importantly, as observed in our analysis, these baseline disparities likely contribute significantly to the less favorable response trajectories in the C2 cluster. However, these baseline variables failed to yield a model with satisfactory discriminative capability, as reflected in our Model 1.

Building upon the more granular response patterns uncovered by unsupervised longitudinal consensus clustering, we developed a predictive model that integrates early response data at week 2. Unlike traditional responder analyses that focus on a single endpoint at fixed time points, the current predictive model (Model 2) is aimed at forecasting a composite, longitudinal trajectory through 24 weeks, providing an early-warning tool for potential non-response. This approach captures the multidimensional nature of AS, encompassing both subjective patient-reported outcomes and objective markers. The strength of this strategy lies in its simplicity and feasibility, bypassing the complex underlying mechanisms not captured by routinely collected clinical data.

Further efforts are needed to externally validate these results. Integration of pharmacometrics (48) and pharmacogenetics (2) with early response as targets may represent a promising step toward more personalized treatment optimization.

The present study has several limitations. First, the consensus clustering analysis yielded only modest cluster separation, meaning that cluster membership is not strictly unambiguous. Our choice of the least ambiguous 2-cluster solution partially reflects its simplicity and clinical interpretability. The clusters should be viewed as a data-driven characterization of treatment response trajectories, rather than evidence of biologically distinct endotypes. The divergence may also reflect substantial baseline differences, particularly in disease activity and prior TNFi exposure. However, as evidenced by baseline predictive models (6, 45), including our Model 1, these pre-existing differences alone cannot fully account for the heterogeneity in treatment responses, highlighting the necessity of integrating early-response data. Second, given the exploratory nature of this study, the lack of external validation in an independent cohort remains a major limitation. While consensus clustering utilizes resampling to assess internal stability, additional prospective robustness analyses are warranted to confirm the proposed clustering solution. Consequently, the predictive model and accompanying online calculator are intended strictly as hypothesis-generating tools to facilitate future validation and should not be used clinically. Third, the endpoints in AS rely heavily on patient-reported outcomes. Although these patient-centric assessments have been validated extensively (49), including in Chinese populations (50), their inherent subjectivity means that the cluster assignments may be vulnerable to measurement variability when applied in daily practice. Fourth, our study population was restricted to a clinical trial population with active AS fulfilling the modified New York criteria and receiving adalimumab or its biosimilar. While this improves homogeneity, it also limits the generalizability of the observed trajectories to the broader, more heterogeneous spectrum of axial spondyloarthritis patients—particularly those with non-radiographic disease. Whether the early-response trajectories remain consistent for responses to other TNFi agents or IL-17 inhibitors also requires further investigation. Finally, although we conducted sensitivity analyses—including multiple imputation and pattern mixture modeling—to confirm the robustness of our findings, the true missingness mechanism cannot be empirically verified. Therefore, the inherent limitations regarding the handling and reporting of data missingness in this *post-hoc* analysis must be acknowledged.

In conclusion, while adalimumab or its biosimilar IBI303 has demonstrated substantial clinical efficacy in active AS, the observed treatment heterogeneity offers an opportunity for patient stratification. Using a data-driven approach, we characterized subgroups with divergent response trajectories to ADA/IBI303 that were evident as early as week 2 and persisted through the 24-week follow-up period. It is possible to recognize these clusters based on data collected within the first two weeks. Future efforts should focus on externally validating these preliminary findings and refining the prospective identification of patients likely to experience less favorable outcomes (such as the C2 cluster).

## Data Availability

The original contributions presented in the study are included in the article/**Supplementary Material**. Further inquiries can be directed to the corresponding authors.

## References

[1] Mauro D Thomas R Guggino G Lories R Brown MA Ciccia F . Ankylosing spondylitis: an autoimmune or autoinflammatory disease? Nat Rev Rheumatol. (2021) 17:387–404. doi: 10.1038/s41584-021-00625-y 34113018

[2] Ortolan A Cozzi G Lorenzin M Galozzi P Doria A Ramonda R . The genetic contribution to drug response in spondyloarthritis: a systematic literature review. Front Genet. (2021) 12:703911. doi: 10.3389/fgene.2021.703911 34354741 PMC8329488

[3] Wen Y Hu Z Xie B Yuan F Xie Z Jiang Y . The trend of targeted therapies in Chinese patients with ankylosing spondylitis: results from a real-life survey. Front Pharmacol. (2021) 12:763707. doi: 10.3389/fphar.2021.763707 34776979 PMC8581396

[4] Curtis JR Winthrop K Bohn RL Suruki R Siegel S Stark JL . The annual diagnostic prevalence of ankylosing spondylitis and axial spondyloarthritis in the United States using Medicare and MarketScan databases. ACR Open Rheumatol. (2021) 3:743–52. doi: 10.1002/acr2.11316 34550648 PMC8593814

[5] Maxwell LJ Zochling J Boonen A Singh JA Veras MMS Tanjong Ghogomu E . TNF‐alpha inhibitors for ankylosing spondylitis. Cochrane Database System Rev. (2015) 4:CD005468. doi: 10.1002/14651858.CD005468.pub2 25887212 PMC11200207

[6] Wang R Dasgupta A Ward MM . Predicting probability of response to tumor necrosis factor inhibitors for individual patients with ankylosing spondylitis. JAMA Netw Open. (2022) 5:e222312. doi: 10.1001/jamanetworkopen.2022.2312 35289857 PMC8924712

[7] MaChado P Landewé R Lie E Kvien TK Braun J Baker D . Ankylosing Spondylitis Disease Activity Score (ASDAS): defining cut-off values for disease activity states and improvement scores. Ann Rheum Dis. (2011) 70:47–53. doi: 10.1136/ard.2010.138594 21068095

[8] Agache I Zemelka-Wiącek M Shamji MH Jutel M . Immunotherapy: state-of-the-art review of therapies and theratypes. J Allergy Clin Immunol. (2022) 150:1279–88. doi: 10.1016/j.jaci.2022.10.007 36328808

[9] U.S. Department of Health and Human Services Food and Drug Administration Center for Drug Evaluation and Research (CDER) Center for Biologics Evaluation and Research (CBER) . Enrichment strategies for clinical trials to support determination of effectiveness of human drugs and biological products guidance for industry (2019). Available online at: https://www.fda.gov/media/121320/download (Accessed November 19, 2025).

[10] van der Heijde D Bellamy N Calin A Dougados M Khan MA van der Linden S . Preliminary core sets for endpoints in ankylosing spondylitis. Assessments in Ankylosing Spondylitis Working Group. J Rheumatol. (1997) 24:2225–9. doi: 10.1016/j.berh.2006.03.011 9375888

[11] Chen Y-H Huang W-N Chen Y-M Lai K-L Hsieh T-Y Hung W-T . The BASDAI cut-off for disease activity corresponding to the ASDAS scores in a Taiwanese cohort of ankylosing spondylitis. Front Med (Lausanne). (2022) 9:856654. doi: 10.3389/fmed.2022.856654 35652077 PMC9149077

[12] Sieper J Poddubnyy D . What is the optimal target for a T2T approach in axial spondyloarthritis? Ann Rheum Dis. (2021) 80:1367–9. doi: 10.1136/annrheumdis-2021-220603 34144966 PMC8522449

[13] Hagenberg J Budde M Pandeva T Kondofersky I Schaupp SK Theis FJ . longmixr: a tool for robust clustering of high-dimensional cross-sectional and longitudinal variables of mixed data types. Bioinformatics. (2024) 40:btae137. doi: 10.1093/bioinformatics/btae137 38485697 PMC10994717

[14] Sieper J Rudwaleit M Baraliakos X Brandt J Braun J Burgos-Vargas R . The assessment of SpondyloArthritis international society (ASAS) handbook: a guide to assess spondyloarthritis. Ann Rheum Dis. (2009) 68:ii1–44. doi: 10.1136/ard.2008.104018 19433414

[15] Garrett S Jenkinson T Kennedy LG Whitelock H Gaisford P Calin A . A new approach to defining disease status in ankylosing spondylitis: the Bath Ankylosing Spondylitis Disease Activity Index. J Rheumatol. (1994) 21:2286–91. 7699630

[16] Flouri I Goutakoli P Repa A Bertsias A Avgoustidis N Eskitzis A . Distinct long-term disease activity trajectories differentiate early on treatment with etanercept in both rheumatoid arthritis and spondylarthritis patients: a prospective cohort study. Rheumatol Int. (2024) 44:249–61. doi: 10.1007/s00296-023-05455-7 37815625 PMC10796740

[17] Portier E Benattar L Resche-Rigon M Dougados M Gossec L Molto A . Different disease activity trajectories in early axial spondyloarthritis lead to significantly different long-term outcomes: a trajectory-based analysis of the DESIR cohort over 10 years. RMD Open. (2024) 10:e004910. doi: 10.1136/rmdopen-2024-004910 39710430 PMC11664343

[18] Molto A Tezenas Du Montcel S Wendling D Dougados M Vanier A Gossec L . Disease activity trajectories in early axial spondyloarthritis: results from the DESIR cohort. Ann Rheum Dis. (2017) 76:1036–41. doi: 10.1136/annrheumdis-2016-209785 27888172

[19] van der Heijde D Schiff MH Sieper J Kivitz AJ Wong RL Kupper H . Adalimumab effectiveness for the treatment of ankylosing spondylitis is maintained for up to 2 years: long-term results from the ATLAS trial. Ann Rheum Dis. (2009) 68:922–9. doi: 10.1136/ard.2007.087270 18701556 PMC2674550

[20] Sieper J van der Heijde D Dougados M Brown LS Lavie F Pangan AL . Early response to adalimumab predicts long-term remission through 5 years of treatment in patients with ankylosing spondylitis. Ann Rheum Dis. (2012) 71:700–6. doi: 10.1136/annrheumdis-2011-200358 22128084 PMC3329233

[21] Xu H Li Z Wu J Xing Q Shi G Li J . IBI303, a biosimilar to adalimumab, for the treatment of patients with ankylosing spondylitis in China: a randomised, double-blind, phase 3 equivalence trial. Lancet Rheumatol. (2019) 1:e35–43. doi: 10.1016/S2665-9913(19)30013-X 38229357

[22] van der Linden S Valkenburg HA Cats A . Evaluation of diagnostic criteria for ankylosing spondylitis. A proposal for modification of the New York criteria. Arthritis Rheum. (1984) 27:361–8. doi: 10.1002/art.1780270401 6231933

[23] Monti S Tamayo P Mesirov J Golub T . Consensus clustering: a resampling-based method for class discovery and visualization of gene expression microarray data. Mach Learn. (2003) 52:91–118. doi: 10.1023/A:1023949509487 41886696

[24] Șenbabaoğlu Y Michailidis G Li JZ . Critical limitations of consensus clustering in class discovery. Sci Rep. (2014) 4:6207. doi: 10.1038/srep06207 25158761 PMC4145288

[25] Schulz KF Altman DG Moher D . CONSORT 2010 statement: updated guidelines for reporting parallel group randomised trials. BMJ. (2010) 340:c332. doi: 10.1136/bmj.c332 20332509 PMC2844940

[26] Pedroza C Truong VT . Performance of models for estimating absolute risk difference in multicenter trials with binary outcome. BMC Med Res Methodol. (2016) 16:113. doi: 10.1186/s12874-016-0217-0 27576307 PMC5006411

[27] European, Medicines, Agency . Guideline on missing data in confirmatory clinical trials (2011). Available online at: https://www.ema.europa.eu/en/documents/scientific-guideline/guideline-missing-data-confirmatory-clinical-trials_en.pdf (Accessed November 15, 2025).

[28] Papp KA Fonjallaz P Casset-Semanaz F Krueger JG Wittkowski KM . Approaches to reporting long-term data. Curr Med Res Opin. (2008) 24:2001–8. doi: 10.1185/03007990802215315 18534049 PMC2853234

[29] Ratitch B O’Kelly M Tosiello R . Missing data in clinical trials: from clinical assumptions to statistical analysis using pattern mixture models. Pharm Stat. (2013) 12:337–47. doi: 10.1002/pst.1549 23292975

[30] Steyerberg EW Harrell FE Borsboom GJ Eijkemans MJ Vergouwe Y Habbema JD . Internal validation of predictive models: efficiency of some procedures for logistic regression analysis. J Clin Epidemiol. (2001) 54:774–81. doi: 10.1016/s0895-4356(01)00341-9 11470385

[31] Efron B Tibshirani R . Improvements on cross-validation: the 632+ bootstrap method. J Am Stat Assoc. (1997) 92:548–60. doi: 10.1080/01621459.1997.10474007 37339054

[32] Pencina MJ D’Agostino RB D’Agostino RB Vasan RS . Evaluating the added predictive ability of a new marker: from area under the ROC curve to reclassification and beyond. Stat Med. (2008) 27:157–72. doi: 10.1002/sim.2929 17569110

[33] R Core Team . R: A language and environment for statistical computing (2025). Available online at: https://www.R-project.org/ (Accessed November 15, 2025).

[34] Bove DS Li L Dedic J Kelkhoff D Kunzmann K Lang BM . mmrm: mixed models for repeated measures (2025). Available online at: https://openpharma.github.io/mmrm/ (Accessed November 15, 2025).

[35] Halekoh U Højsgaard S Yan J . The R package geepack for generalized estimating equations. J Stat Softw. (2006) 15/2:1–11. doi: 10.18637/jss.v015.i02

[36] Lenth RV . emmeans: Estimated Marginal Means, aka Least-Squares Means (2025). Available online at: https://CRAN.R-project.org/package=emmeans (Accessed November 15, 2025).

[37] Cho JH Feldman M . Heterogeneity of autoimmune diseases: pathophysiologic insights from genetics and implications for new therapies. Nat Med. (2015) 21:730–8. doi: 10.1038/nm.3897 26121193 PMC5716342

[38] Aggarwal R Ringold S Khanna D Neogi T Johnson SR Miller A . Distinctions between diagnostic and classification criteria? Arthritis Care Res (Hoboken). (2015) 67:891–7. doi: 10.1002/acr.22583 25776731 PMC4482786

[39] Rudwaleit M van der Heijde D Landewé R Listing J Akkoc N Brandt J . The development of Assessment of SpondyloArthritis international Society classification criteria for axial spondyloarthritis (part II): validation and final selection. Ann Rheum Dis. (2009) 68:777–83. doi: 10.1136/ard.2009.108233 19297344

[40] Dasgupta B Salvarani C Schirmer M Crowson CS Maradit-Kremers H Hutchings A . Developing classification criteria for polymyalgia rheumatica: comparison of views from an expert panel and wider survey. J Rheumatol. (2008) 35:270–7. 18050370

[41] Nam EJ Lee WK . Early improvements in disease activity indices predict long-term clinical remission suggested by the treat-to-target strategy in patients with ankylosing spondylitis receiving TNF-α inhibitor treatment. J Clin Med. (2021) 10:4279. doi: 10.3390/jcm10184279 34575390 PMC8469764

[42] Ward MM Deodhar A Gensler LS Dubreuil M Yu D Khan MA . 2019 update of the American College of Rheumatology/Spondylitis Association of America/Spondyloarthritis Research and Treatment Network recommendations for the treatment of ankylosing spondylitis and nonradiographic axial spondyloarthritis. Arthritis Rheumatol. (2019) 71:1599–613. doi: 10.1002/art.41042 31436036 PMC6764882

[43] Ramiro S Nikiphorou E Sepriano A Ortolan A Webers C Baraliakos X . ASAS-EULAR recommendations for the management of axial spondyloarthritis: 2022 update. Ann Rheum Dis. (2023) 82:19–34. doi: 10.1136/ard-2022-223296 36270658

[44] Guler MA Celik OF Ayhan FF . The important role of central sensitization in chronic musculoskeletal pain seen in different rheumatic diseases. Clin Rheumatol. (2020) 39:269–74. doi: 10.1007/s10067-019-04749-1 31446538

[45] Lee S Kang S Eun Y Won H-H Kim H Lee J . Machine learning-based prediction model for responses of bDMARDs in patients with rheumatoid arthritis and ankylosing spondylitis. Arthritis Res Ther. (2021) 23:254–65. doi: 10.1186/s13075-021-02635-3 34627335 PMC8501710

[46] Hu L Ji X Wang Y Man S Liu X Wang L . Underweight and obesity are strong predictors of clinical outcomes in patients with ankylosing spondylitis: data from the smart-phone SpondyloArthritis management system. Ther Adv Musculoskelet Dis. (2021) 13:1759720X211030792. doi: 10.1177/1759720X211030792 34345253 PMC8280843

[47] Navarro-Compán V Plasencia-Rodríguez C de Miguel E Diaz Del Campo P Balsa A Gratacós J . Switching biological disease-modifying antirheumatic drugs in patients with axial spondyloarthritis: results from a systematic literature review. RMD Open. (2017) 3:e000524. doi: 10.1136/rmdopen-2017-000524 29071119 PMC5640114

[48] Peck RW . Precision medicine is not just genomics: the right dose for every patient. Annu Rev Pharmacol Toxicol. (2018) 58:105–22. doi: 10.1146/annurev-pharmtox-010617-052446 28961067

[49] Landewé R van Tubergen A . Clinical tools to assess and monitor spondyloarthritis. Curr Rheumatol Rep. (2015) 17:47. doi: 10.1007/s11926-015-0522-3 26063534 PMC4464370

[50] Lin Z Gu J He P Gao J Zuo X Ye Z . Multicenter validation of the value of BASFI and BASDAI in Chinese ankylosing spondylitis and undifferentiated spondyloarthropathy patients. Rheumatol Int. (2011) 31:233–8. doi: 10.1007/s00296-009-1313-9 20012866 PMC3025108

