# Supplementary material for: Early divergence of treatment response trajectories to adalimumab or its biosimilar in active ankylosing spondylitis: consensus clustering analysis of a randomized controlled trial
Source: Front Immunol. 2026 Jun 23;17:1804751. doi: 10.3389/fimmu.2026.1804751 (PMC13337383; doi:10.3389/fimmu.2026.1804751)
Supplement: Supplementary file 1 [file DataSheet1.pdf]

Supplementary Material

Content

1 Data Missingness and Imputation 3

1.1 Data Missing Pattern 3

1.2 Data Imputation 3

1.3 Consistence of results among the imputed datasets 5

2 Consensus Clustering 7

3 Development and Internal Validation of Predictive Model of Cluster C2 12

3.1 LASSO for Variable Selection 14

3.2 Model Evaluation 16

3.3 Model Presentation 18

References 20

Figures

Supplementary Figure 1. Data Missingness Pattern Stratified by Cluster
Supplementary Figure 2. Data Missingness Pattern Stratified by Cluster
Supplementary Figure 3. Scatterplots of BASFI over time with different δ values
Supplementary Figure 4. Impact of different δ parameters in the Pattern Mixture Model Imputation on the estmates and confidence intervals of BASDAI change from baseline
Supplementary Figure 5. Impact of different δ in Pattern Mixture Model Imputation on the estmates and confidence intervals of ASDAS-MI response rate
Supplementary Figure 6. Other Diagnostic Metrics of Clustering Solution.
Supplementary Figure 7. Agreements of clustering membership among different choice of functional forms (A) and degree of freedom in the spline model (B).
Supplementary Figure 8. Agreements of Cluster Membership Among the Original and Imputed Datasets
Supplementary Figure 9. Diagnostic plots informing optimal number of clusters when the 10 original variables were selected as inputs of the consensus clustering algorithm (A) and cluster membership agreement with those constructed from the first two principal components (B).
Supplementary Figure 10. Time-course of each variables in consensus cluster 1 (C1) and cluster 2 (C2)
Supplementary Figure 11. K = 4 solution. Relationship between membership of K =2 solution (A) and trajectory plot of continuous outcomes (B)
Supplementary Figure 12. Diagnostic plots informing optimal number of clusters after excluding data within 2 weeks (A) and cluster membership agreement with those constructed from the full data (B).
Supplementary Figure 13. Bootstrap-based LASSO variable selection for Model 1
Supplementary Figure 14. Bootstrap-based LASSO variable selection for Model 2
Supplementary Figure 15. Model Perfomance Plots, including ROC curves (A), Decision Curves (B) and Calibration Plots (C) of models based on baseline variables only (Model 1) and on baseline and week-2 data (Model 2)
Supplementary Figure 16. Predictive Model of Cluster C2 based on baseline and week 2 data, presented in Nomogram (A) and online shiny-based calculator (B)

Tables

Supplementary Table 1. Sensitivity Analysis of Different Model Configuration
Supplementary Table 2. Candidate variables for model construction with assessment of near-zero variance
Supplementary Table 3. Performance of Model based on basline data only (Model 1) and on basline plus week-2 data(Model 2)
Supplementary Table 4. Coeffients of Prediction Model of Cluster C2

1 Data Missingness and Imputation

1.1 Data Missing Pattern

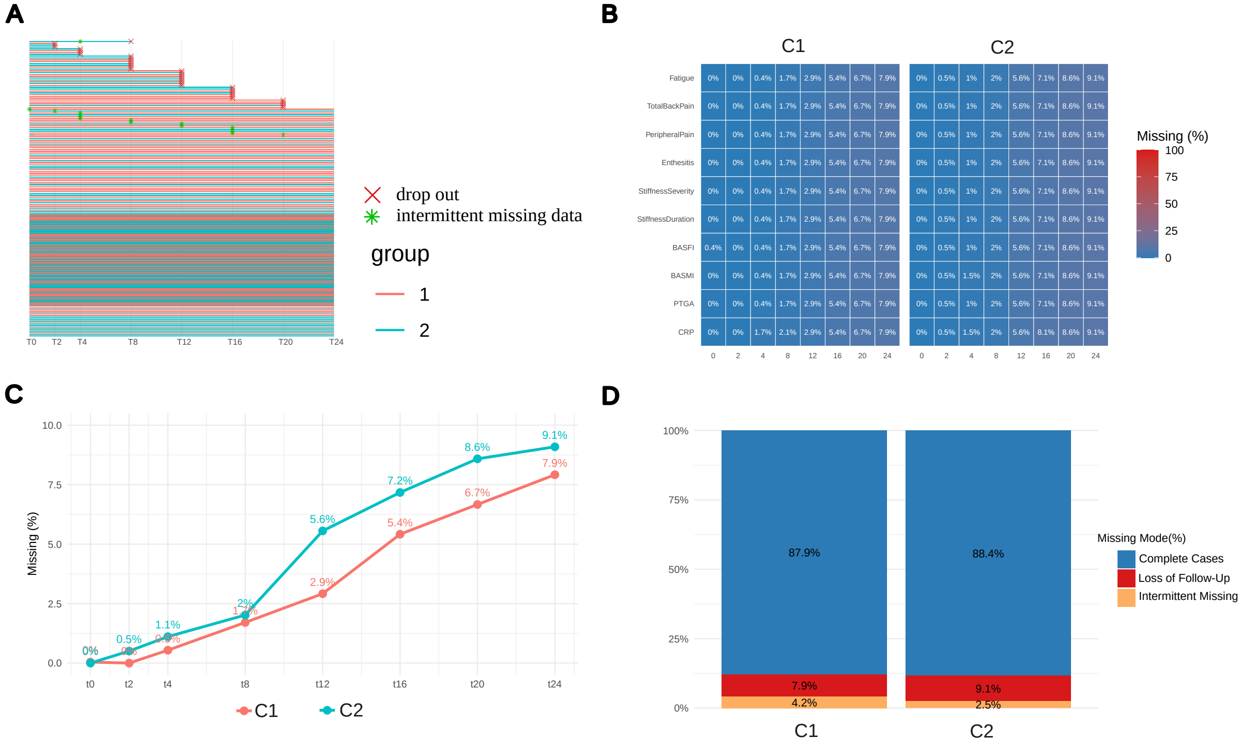

Supplementary Figure 1. Data Missingness Pattern Stratified by Cluster Individual-level missing pattern depicted by swim plot(A), Variable-level missing proportion (B), Cluster-level missing proportion at each visit(C) and overall classification of missingnes (D) were presented.

1.2 Data Imputation

Our main data imputation methods are consistent with the main stream publications (1–3): non-responder imputation (NRI) for binary outcomes and mixed-effects models for repeated measures (MMRM) for continuous outcomes.

Besides, we also performed sensitivity analysis by testing alternative imputation method, i.e., the δ-adjustment approach in the pattern mixture modelling (PMM) framework (4).Briefly, this two-step approach first generates multiple imputed datasets with multiple imputation (MI) under MAR and then adds an offset parameter δ to each of the imputed variables, as detailed in the online tutorial (5).

- We used multiple imputation by chained equations (MICE) with the mice package (6) for the MI step, with the following parameters: Number of imputed datasets: 10 (according to the rule of thumb that m imputations adequate for m% missing rate (7)), Maximum iterations: 50; Imputation method: Predictive Mean Matching (PMM); Missing data with the decreasing proportion of missingness were imputed sequentially (Monotone visit sequence); All variables were used as predictors to impute any missing value, as recommended (8).

- In the δ step, we tested a range of clinically plausible values to mimic the situation of drop-outs due to worsening of disease activity: For outcome variables bounded by 0 and 10, such as BASFI, δ values of 1.5, 3.0, 4.5, 6.0 were tested; For C-reactive protein levels, ln(δ) was added at the log scale, so that δ of 1.5 means 1.5 times the original value. The minimum of the upper limit of that variable and δ -added value was determined as the final imputed value.

- For each δ , 10 imputed datasets were anaylzed separately and then combined according to the Rubin's rule (9) in appropriate scales (e.g., logarithm for odds ratios).

The adequacy of the imputation process in the MI step was checked by traceplot (**Supplementary Figure 2**) and final results illustrated by scatterplot of the observed and imputed values (**Supplementary Figure 3**).

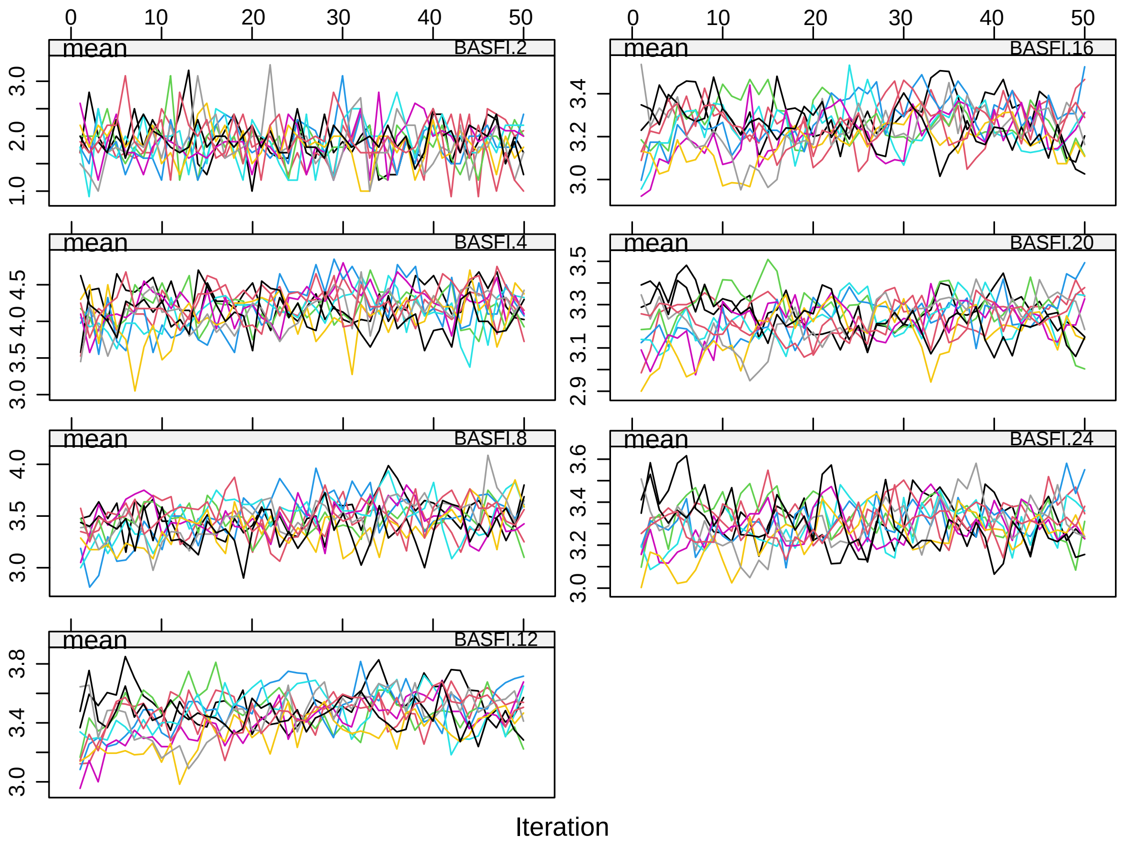

Supplementary Figure 2. Trace plots for multiple imputation convergence. BASFI at week 2 4, 8, 12,16,20, 24 as an example.

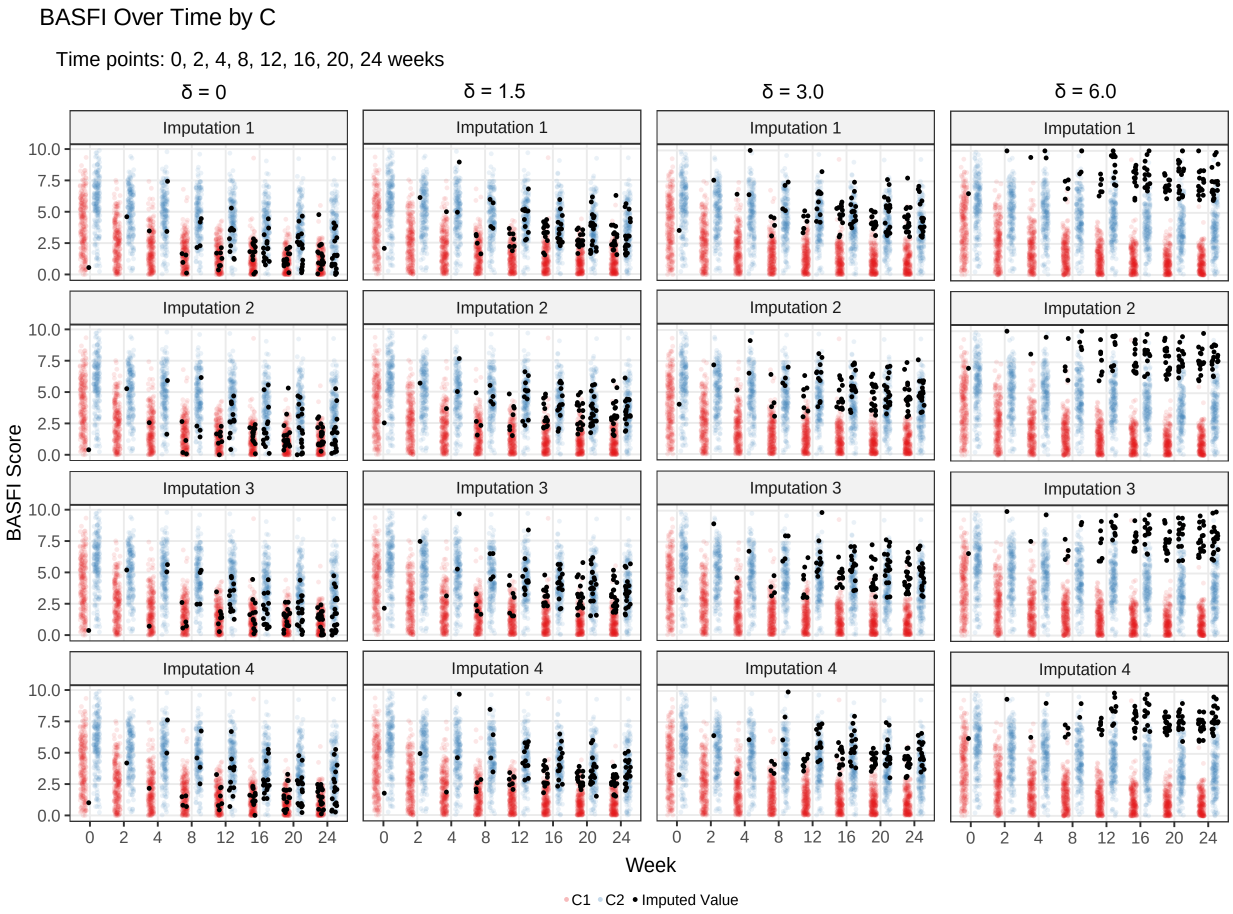

Supplementary Figure 3. Scatterplots of BASFI over time with different δ values. Only the first 4 imputed datasets of each δ were presented for brevity; the remaining imputed datasets exhibited similar patterns.

1.3 Consistence of results among the imputed datasets

We assessed the longitudinal response curves accross the original and imputed datasets, to obtain the following observations:

- PMM with δ values of 1.5 to 6.0 (a rather wide range magnitude of disease progression assumed to the drop-outs) had almost ignorable effects on both continuous and binary outcomes;
- NRI generally provided the most conservative estimates (lower bounds) of binary response rate.

Examples were presented in **Supplementary Figure 4** and **Supplementary Figure 5.** These sensitivity analysis support the robustness of the main analysis with regards to the choice of imputation methods.

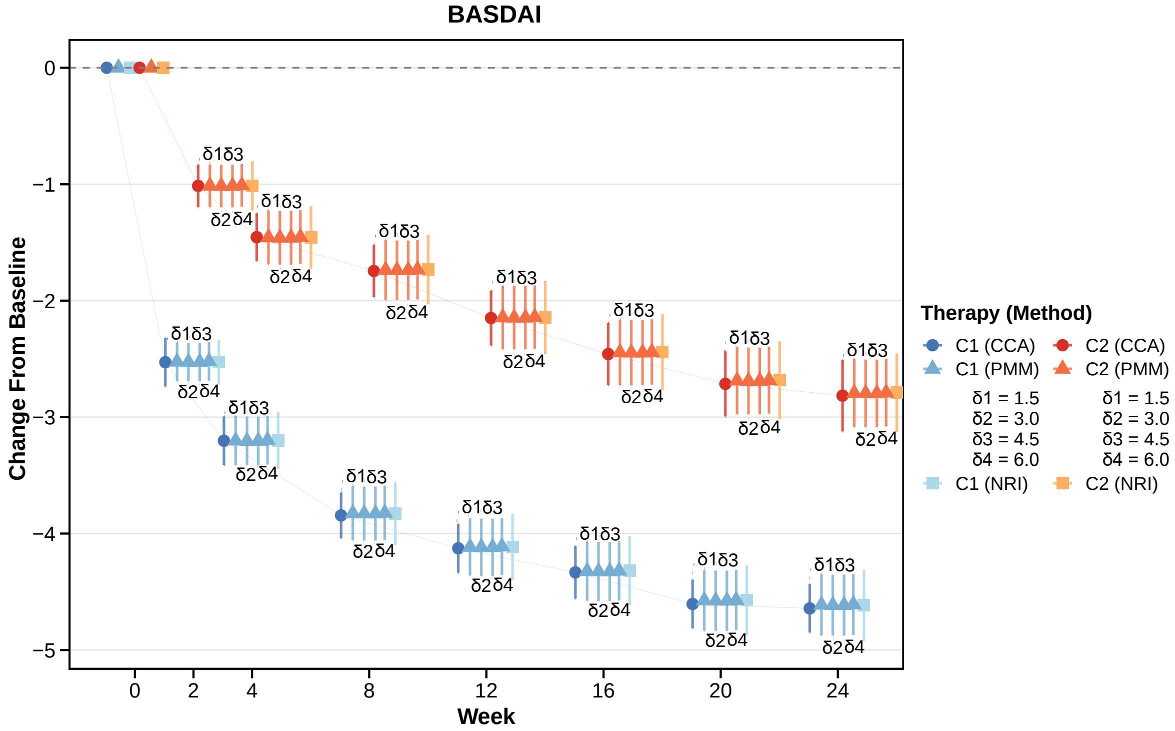

Supplementary Figure 4. Impact of different δ parameters in the Pattern Mixture Model Imputation on the estmates and confidence intervals of BASDAI change from baseline

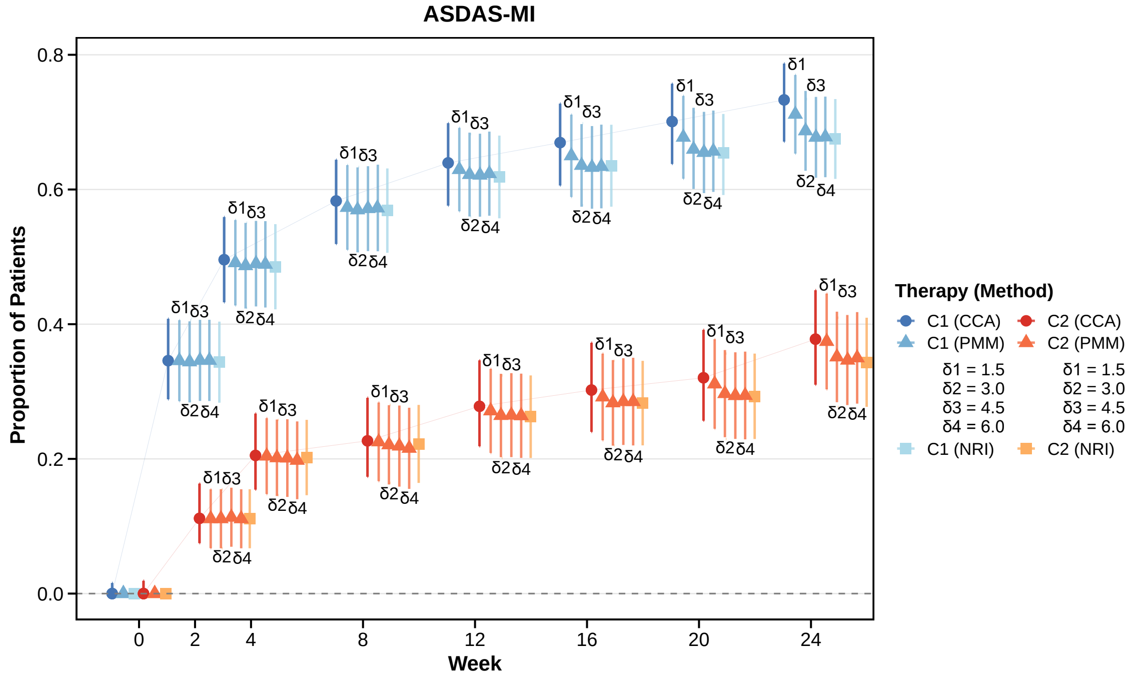

Supplementary Figure 5. Impact of different δ in Pattern Mixture Model Imputation on the estmates and confidence intervals of ASDAS-MI response rate

2 Consensus Clustering

We performed consensus clustering to identify clinically meaningful patient subgroups. This robust clustering approach aggregates multiple clustering results obtained from resampled datasets, enhancing the stability and reproducibility of identified clusters (10).

For each of the 10 core longitudinal variables, we fitted generalized additive mixed models using flexmix::FLXMRmgcv(), with smoothing splines (s(week, k = 4)) to capture nonlinear temporal trends. Parameters for the consensus clustering algorithm included: 50 bootstrap iterations, and 80% patient sampling per iteration (pItem = 0.8). Cluster solutions evaluated ranged from 2 to 5 clusters. Hierarchical clustering with Ward's minimum variance (final_linkage = "ward.D2") were utilized that minimizes total within-cluster variance at each merging step.

The optimal number of clusters (K) was determined following the guidance (10)**.** Briefly, the K with "step-like" shape, i.e., abrupt change of value around 0 and 1 in the cumulative distribution function (CDF plot) and that maximizes the relative increase in the area under the CDF was selected (the "delta area" plot). The item-consensus plots and cluster-consensus plots quantifiy the individual-level and cluster-level consensus of belonging to the same cluster (1 for perfect consensus). As shown in the maintext, clustering number of 2 represented the most robust and reproducible clustering solution. Additional diagnostic metrics are provided below (**Supplementary Figure 6**).

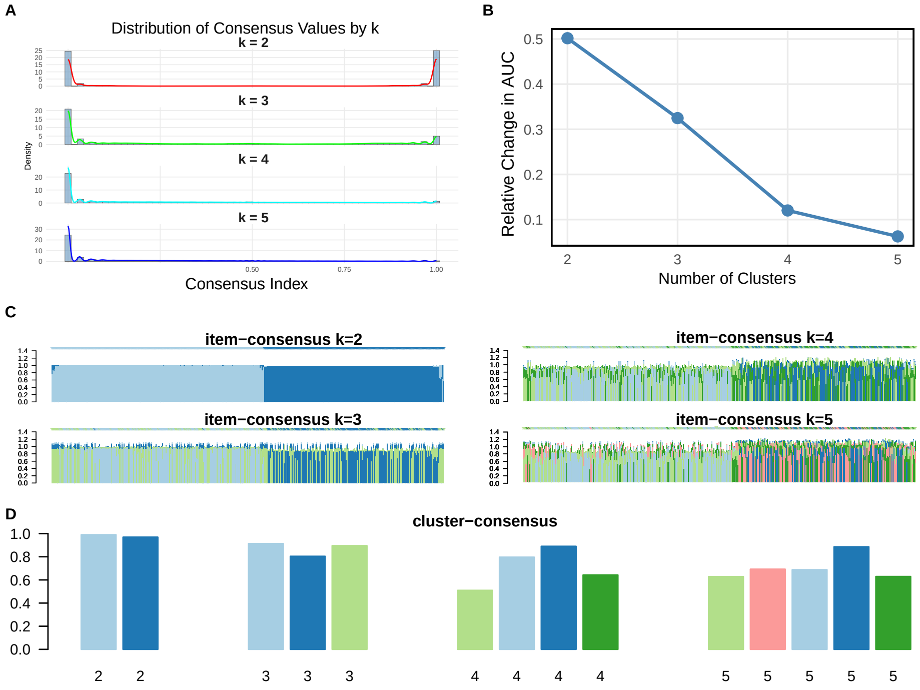

Supplementary Figure 6 Other diagnostic metrics. The histograms of the consensus index values (A), offers an intuitive view of the near-0 and near-1 concentration.The delta area plot (B), which illustrates the relative changes in the area under the CDF curves, levels off at K = 2, indicating diminishing returns in cluster stability when the cohort is divided into more than 2 clusters. The item-consensus plot (C) and the cluster-consensus plot (D) further quantify the individual and cluster-averaged probabilities of co-membership, demonstrating a high degree of individual-level and cluster-level consensus for the 2-cluster solution.

Results of the 10 imputed datesets, original variables vs. principal components as input, as well as several alternative model configurations were also explored, and remained generally consistent with the initial model specification (**Supplementary Table 1, Supplementary Figure 7-8**)

Supplementary Table 1. Sensitivity Analysis of Different Model Configuration

Model	Formula	Agreement with the main model
Linear	~ week | PID	94.7%
Quadratic	~ poly(week, 2) | PID	95.4%
Cubic	~ poly(week, 3) | PID	96.8%
Spline_dof = 1	~ s(week, k = 1) | PID	98.4%
Spline_dof = 2	~ s(week, k = 2) | PID	98.4%
Spline_dof = 3	~ s(week, k = 3) | PID	98.4%
Main (Spline_dof = 4)	~ s(week, k = 4) | PID	100%
Spline_dof = 5	~ s(week, k = 5) | PID	98.9%
Spline_dof = 6	~ s(week, k = 6) | PID	98.4%
Square Polynoimal	~ I(sqrt(week)) + I(week) | PID	96.8%
Logarithm Polynoimal	~ I(log(week + 0.1)) + I(week) | PID	96.3%

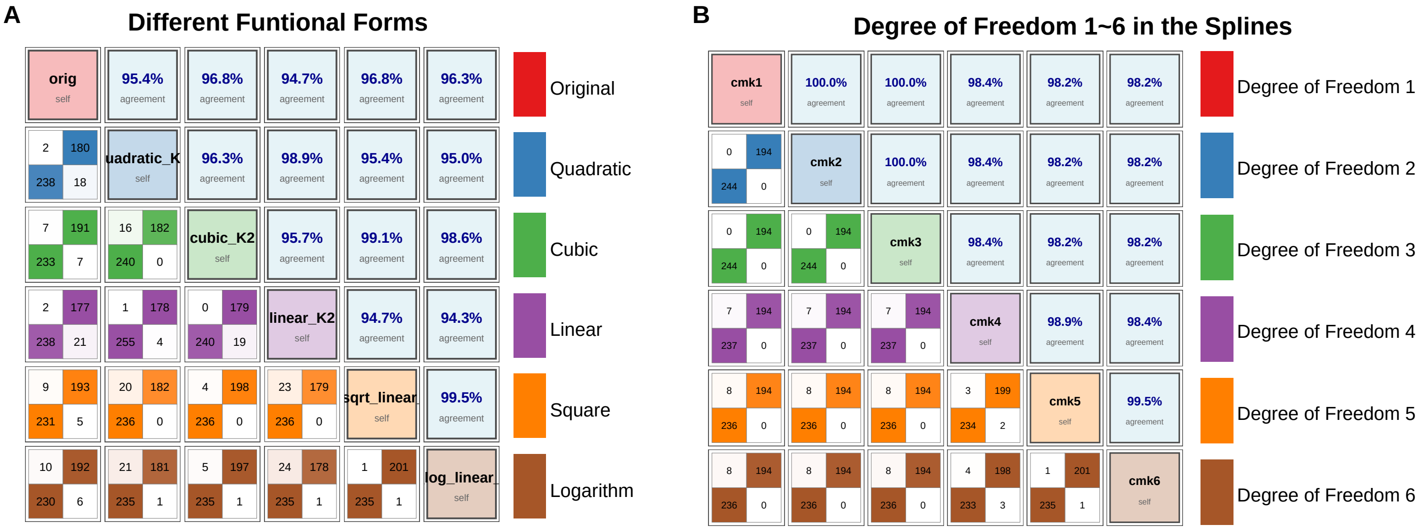

Supplementary Figure 7. Agreements of clustering membership among different choice of functional forms (A) and degree of freedom in the spline model (B). Upper triangle cells report the pairwise percentage agreement, i.e., proportion of samples assigned to the same cluster, between methods intersecting at that cell. Lower triangular panels present standard confusion matrices, where cell values indicate absolute counts.

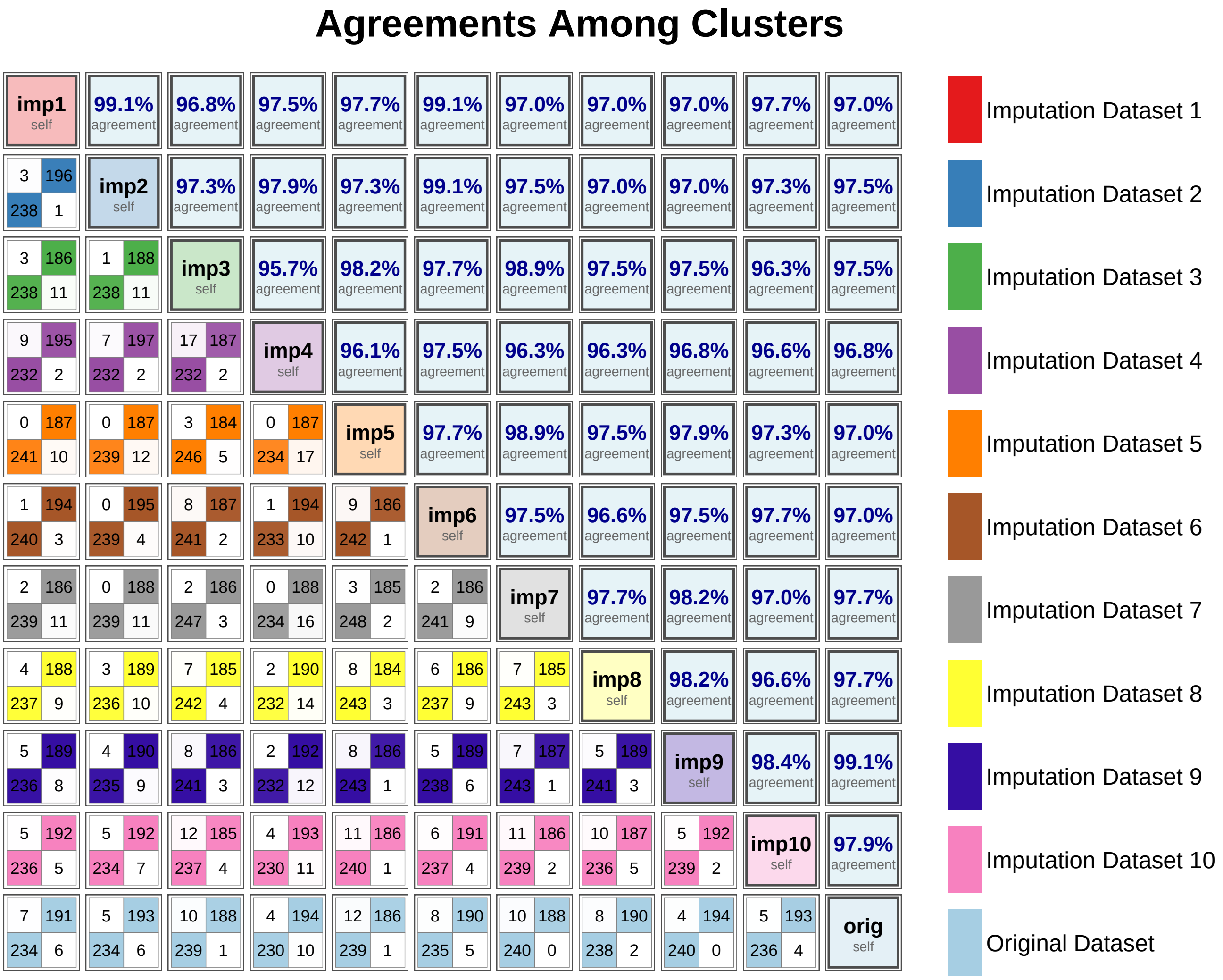

Supplementary Figure 8. Agreements of Cluster Membership Among the Original and Imputed Datasets

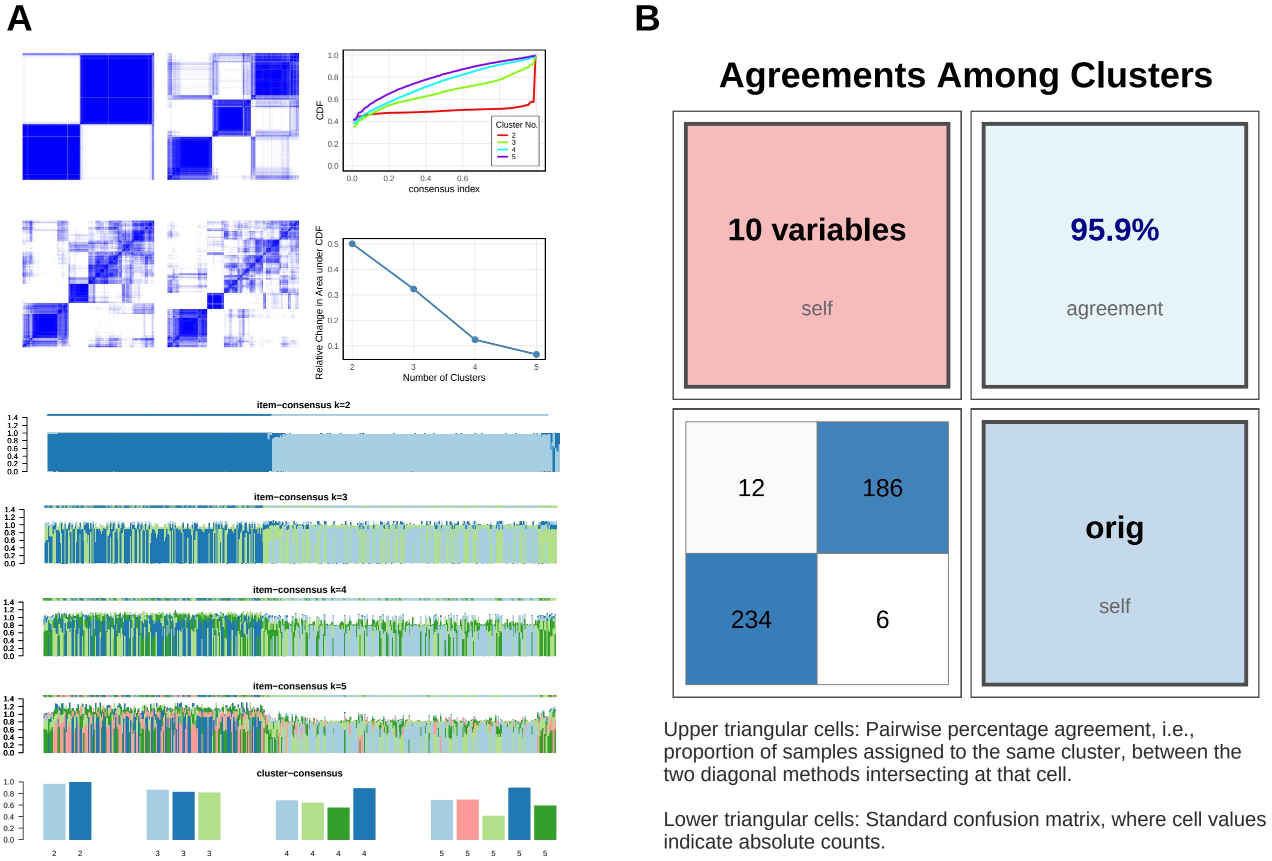

Supplementary Figure 9. Diagnostic plots informing optimal number of clusters when the 10 original variables were selected as inputs of the consensus clustering algorithm (A) and cluster membership agreement with those constructed from the first two principal components (B).

Trajectory of 10 variabes in each cluster (consensus cluster 1 and 2, C1 and C2, number of cluster K = 2), as well as the K=4 solution were depicted in **Supplementary Figure 10 and Supplementary Figure 11, respectively.**

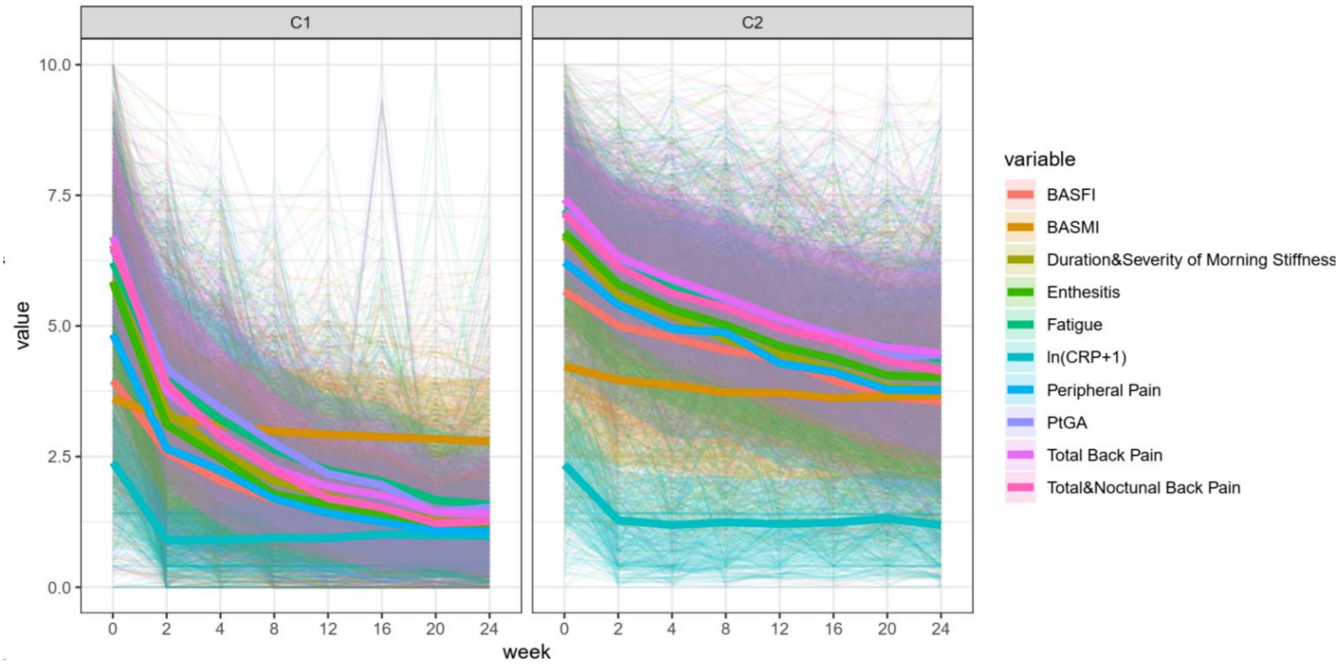

Supplementary Figure 10. Time-course of each variables in consensus cluster 1 (C1) and cluster 2 (C2)

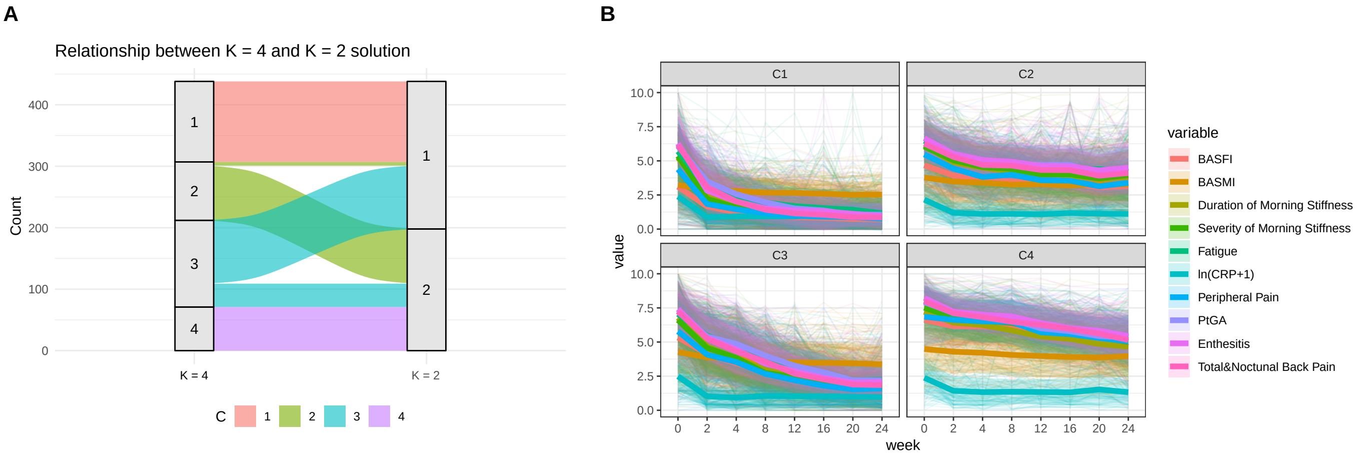

Supplementary Figure 11. K = 4 solution. Relationship between membership of K =2 solution (A) and trajectory plot of continuous outcomes (B)

3 Development and Internal Validation of Predictive Model of cluster C2

We aimed to develop and internally validate a model to predict **cluster C2**, a phenotype defined by unfavorable responses pattern based on consensus clustering of 10 core response variables.

To avoid predictor-outcome overlap, we re-constructed the **clusters** using data beyond week 2. The optimal number of clusters was 2 (**Supplementary Figure 12A**). The resulting cluster membership had 95.6% agreement (Accuracy) with the initial clustering (**Supplementary Figure 12B**), confirming the robustness of the proposed **clusters**.

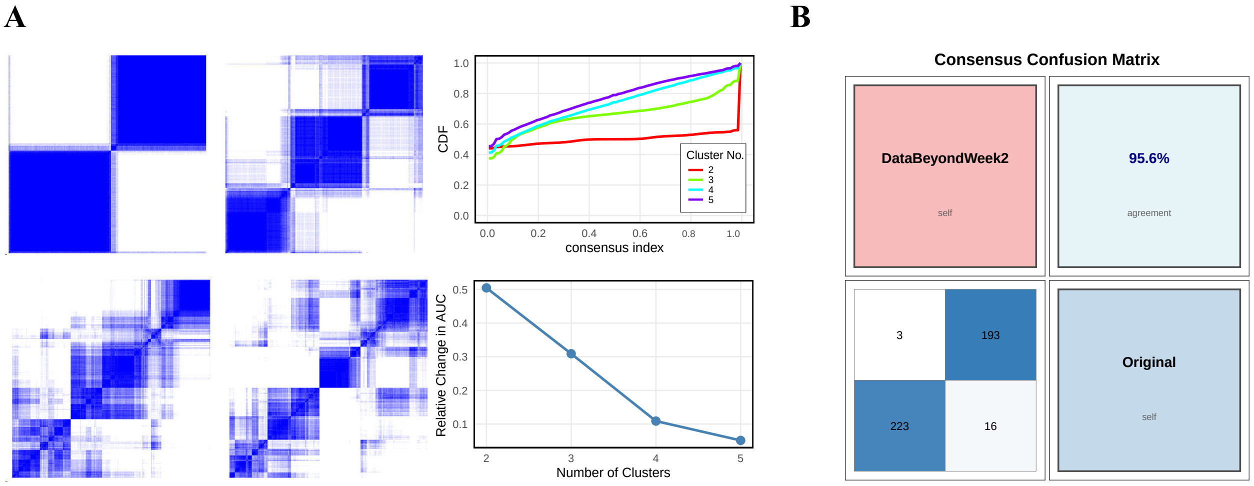

Supplementary Figure 12. Diagnostic plots informing optimal number of clusters after excluding data within 2 weeks (A) and cluster membership agreement with those constructed from the full data (B).

Candidate predictors included data collected at baseline and week 2. These included 20 baseline characteristics and 10 variables measured at baseline as well as week 2. Preprocessing of data with near zero variance filtering was performed (11) (**Supplementary Table 2**).

Supplementary Table 2. Candidate variables for model construction with assessment of near-zero variance

Variable	FreqRatio	PercentUnique	NZV
**Baseline**			
Age	1.0	9.4	FALSE
Sex	4.8	0.5	FALSE
Weight	1.1	27.9	FALSE
Height	1.6	8.9	FALSE
BMI	1.3	32.6	FALSE
Smoking	2.6	0.5	FALSE
Diabetes	72.0	0.5	TRUE
Hypertension	12.7	0.5	FALSE
Cerebrovascular Diseases	145.0	0.5	TRUE
Renal Diseases	61.6	0.5	TRUE
Hyperlipidemia	20.9	0.5	TRUE
Hyperuremia	32.7	0.5	TRUE
Uveitis	18.9	0.5	FALSE
HLA-B27	10.2	0.5	FALSE
Symptom Duration	1.4	6.6	FALSE
Diagnosis Duration	1.1	5.7	FALSE
Previous Methotrexate	2.4	0.5	FALSE
Previous SASP	1.7	0.5	FALSE
Previous Steroids	7.8	0.5	FALSE
Previous TNFi	1.4	0.5	FALSE
**Baseline and Week 2**			
Enthesitis	1.1	18.7	FALSE
Fatigue	1.1	17.1	FALSE
PTGA	1.1	15.5	FALSE
Peripheral Pain	1.2	21.7	FALSE
BASFI	1.3	79.0	FALSE
BASMI	1.2	16.0	FALSE
CRP	1.6	87.9	FALSE

Stiffness Duration	1.1	18.9	FALSE
Stiffness Severity	1.0	17.6	FALSE
Total Back Pain	1.1	15.5	FALSE

freqRatio: ratio of frequencies of the most common value to the second most common value; percentUnique: p ercentage of unique data points out of total number of samples; zeroVar: indicator of zero variance; NZV: indi cator of near-zero variance.

We considered two multi-variable models:

- **Model 1**: Predictors based on data of baseline only
- **Model 2**: Predictors based on data of baseline and week 2

According to the rule of thumb of 10 or more events per variable (EPV) to ensure stable parameter, the model's complexity was constrained to 19 degrees of freedom (192 cases of **cluster C2** in our dataset). We therefore only explored simple logistic regression model, which also offers the additional advantages of greater interpretability and readiness for future external model validation. We used the Least Absolute Shrinkage and Selection Operator (LASSO) to inform variable selection.

3.1 LASSO for Variable Selection

We performed bootstrap-based variable selection, due to its established advantages over split-sample approaches (12,13). A total of 200 bootstrap resamples were drawn with replacement from the original dataset. For each bootstrap sample, LASSO logistic regression models were fitted across a grid of 100 λ values ranging from 0.001 to 0.1. The optimal λ was selected using the one-standard-error (1-SE) criteria, which identifies the largest λ (i.e., the most penalized model) whose mean AUC in the out-of-bag (OOB) sample is within one standard deviation of the maximum mean OOB AUC. The tuning process was visualized with bootstrapped AUC (mean ± SD) plotted against log(λ)(**Supplementary Figure 13A, 14A**). Variables with non-zero regression coefficients at the selected λ were retained as predictors in the final parsimonious model. The frequency of these variables selected across 200 bootstrap replicates were plotted (**Supplementary Figure 13B, 14B**). Then a LASSO model was refitted on the full dataset using the optimal λ, with coefficient paths plotted as a function of log(λ) (**Supplementary Figure 13C, 14C**).

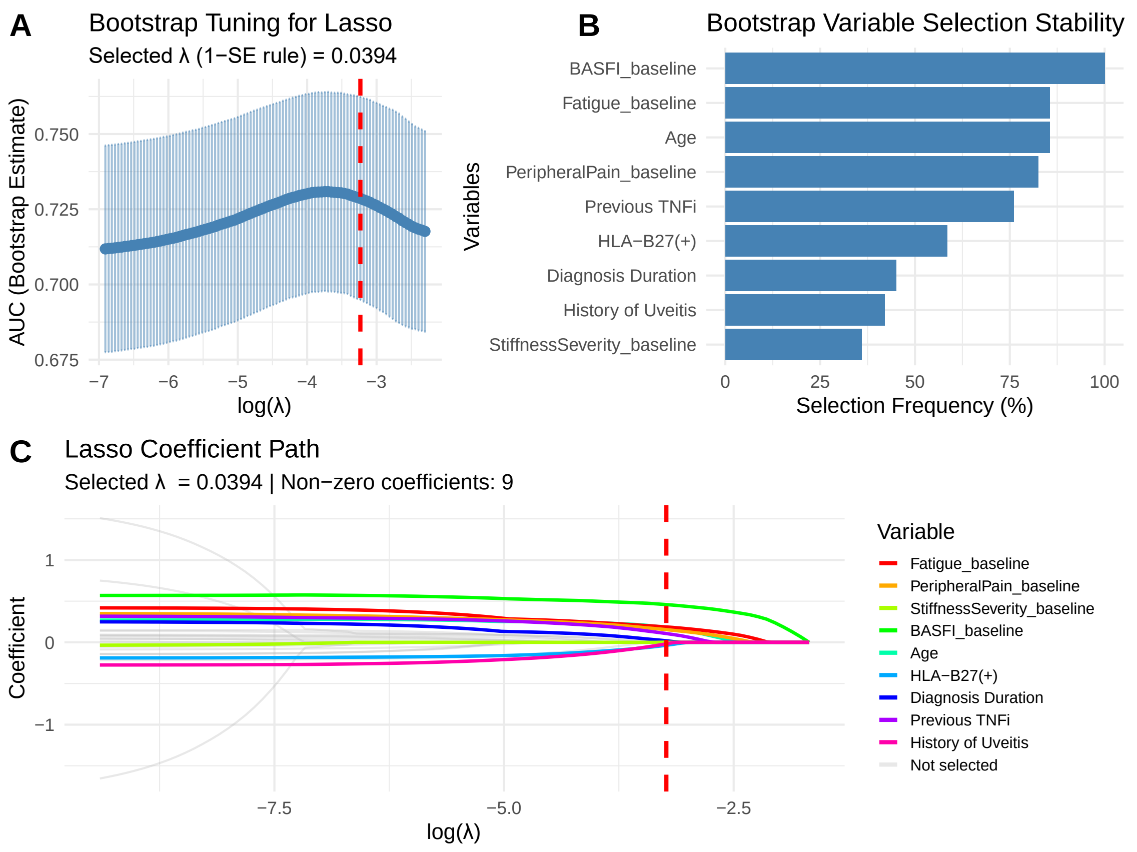

Supplementary Figure 13. Bootstrap-based LASSO variable selection for Model 1

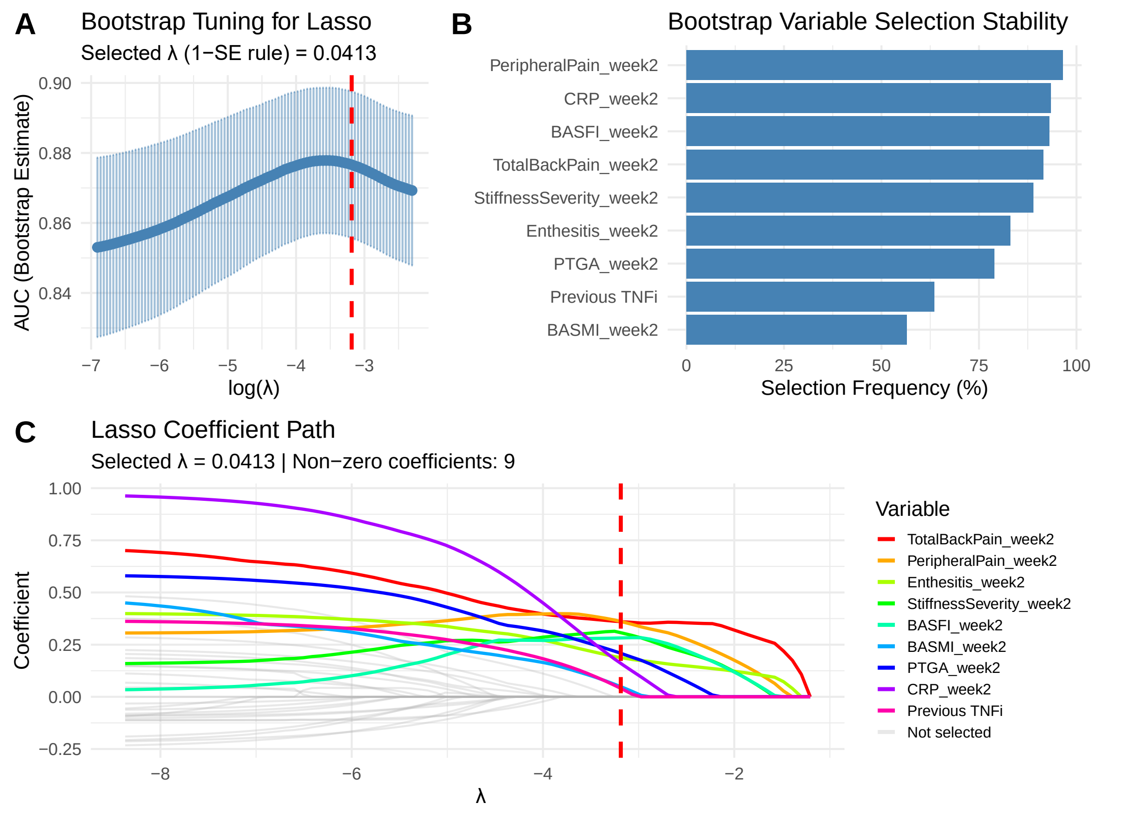

Supplementary Figure 14. Bootstrap-based LASSO variable selection for Model 2

3.2 Model Evaluation

To internally validate the model and corrrect for over-optimism, the following aggregated metrics was estimated using 200 bootstrap resample, including the apparent AUC, AUC in the OOB sample (Bootstrap AUC), optimism-corrected AUC (0.632+ estimator) (13) for discrimination, the Brier Score and scaled Brier Score for overall accuracy, calibration slope, calibration intercept, integrated calibration index (ICI), median and 90th percentile absolute errors (E50 and E90) for calibration (**Supplementary Table 3** and **Supplementary Figure 15**). These calculations were mainly implemented with the R package pminternal (14), designed dedicately for less-biased internal validation of clinical prediction models of binary outcomes, especially with bootstrap methods (15).

Model performance of Model 1 and Model 2 was compared using continuous (category-free) net reclassification improvement (NRI) and integrated discrimination improvement (IDI) (16) (**Supplementary Table 3**).

Supplementary Table 3. Performance of Model based on basline data only (Model 1) and on basline plus week-2 data(Model 2)

Performance Metric	Model 1	Model 2
Apparent AUC	0.764	0.893
Bootstrap AUC (95% CI)	0.722 (0.654, 0.790)	0.866 (0.816, 0.911)
AUC (optimism-corrected)	0.733 (0.703, 0.784)	0.880 (0.859, 0.915)
Brier Score (95% CI)	0.212 (0.211, 0.214)	0.147 (0.146, 0.149)
Scaled Brier (95% CI)	0.144 (0.138, 0.150)	0.406 (0.399, 0.413)
Calibration Slope (95% CI)	1.325 (1.250, 1.459)	1.362 (1.316, 1.495)
Calibration Intercept (95% CI)	0.056 (-0.007, 0.118)	0.050 (-0.001, 0.106)
ICI (95% CI)	0.0159 (0.0127, 0.0201)	0.0304 (0.0276, 0.0335)
E50 (Median Absolute Error, 95% CI)	0.0160 (0.0116, 0.0209)	0.0324 (0.0280, 0.0376)
E90 (90th Percentile Absolute Error, 95% CI)	0.0279 (0.0224, 0.0356)	0.0503 (0.0471, 0.0534)
Selected Variables (n)	9	9
**Comparison (Model_2 vs. Model_1)**		
Continuous NRI		0.980 (0.978, 0.982)
IDI		0.2327 (0.2322, 0.2332)

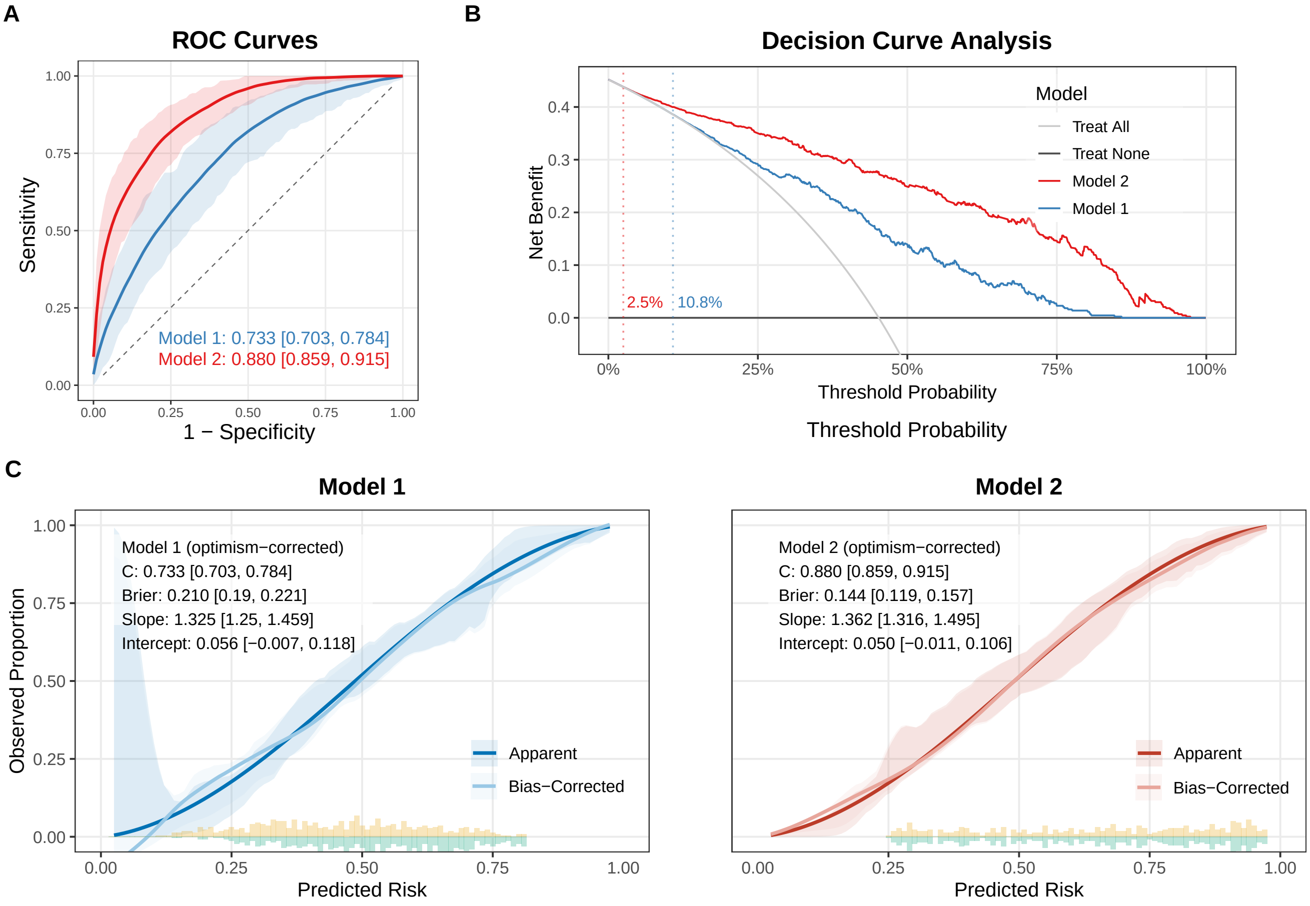

Supplementary Figure 15. Model Perfomance Plots, including ROC curves (A), Decision Curves (B) and Calibration Plots (C) of models based on baseline variables only (Model 1) and on baseline and week-2 data (Model 2)

3.3 Model Presentation

Given the good internal performance of Model 2, the 9 retained variables with non-zero coefficient of Model 2 were used to fit a multivariable logistic regression model, given its feasibility for future idenpendent external validatioon. To facilitate this process, the coefficients of each predictor of the prediction model (**Supplementary Table 4**), a nomogram and a shiny-based web application (**Supplementary Figure 16**) were presented.

Supplementary Table 4. Coeffients of Prediction Model of cluster C2

Variable	β	OR (95% CI)	Wald Z	P-value
Baseline				
Prior TNFi use	0.691	1.996 (1.158-3.441)	2.49	0.013
Week-2				
Total Back Pain	0.189	1.208 (0.908-1.607)	1.30	0.193
Peripheral Pain	0.209	1.232 (1.021-1.486)	2.18	0.029
Enthesitis	0.159	1.172 (0.918-1.495)	1.27	0.202
Stiffness Severity	0.220	1.246 (1.011-1.534)	2.06	0.039
BASFI	0.126	1.134 (0.932-1.381)	1.26	0.208
BASMI	0.179	1.196 (0.987-1.449)	1.83	0.067
PTGA	0.247	1.280 (0.996-1.643)	1.93	0.053
CRP	0.126	1.135 (1.050-1.227)	3.18	0.001
Intercept				
-6.824				

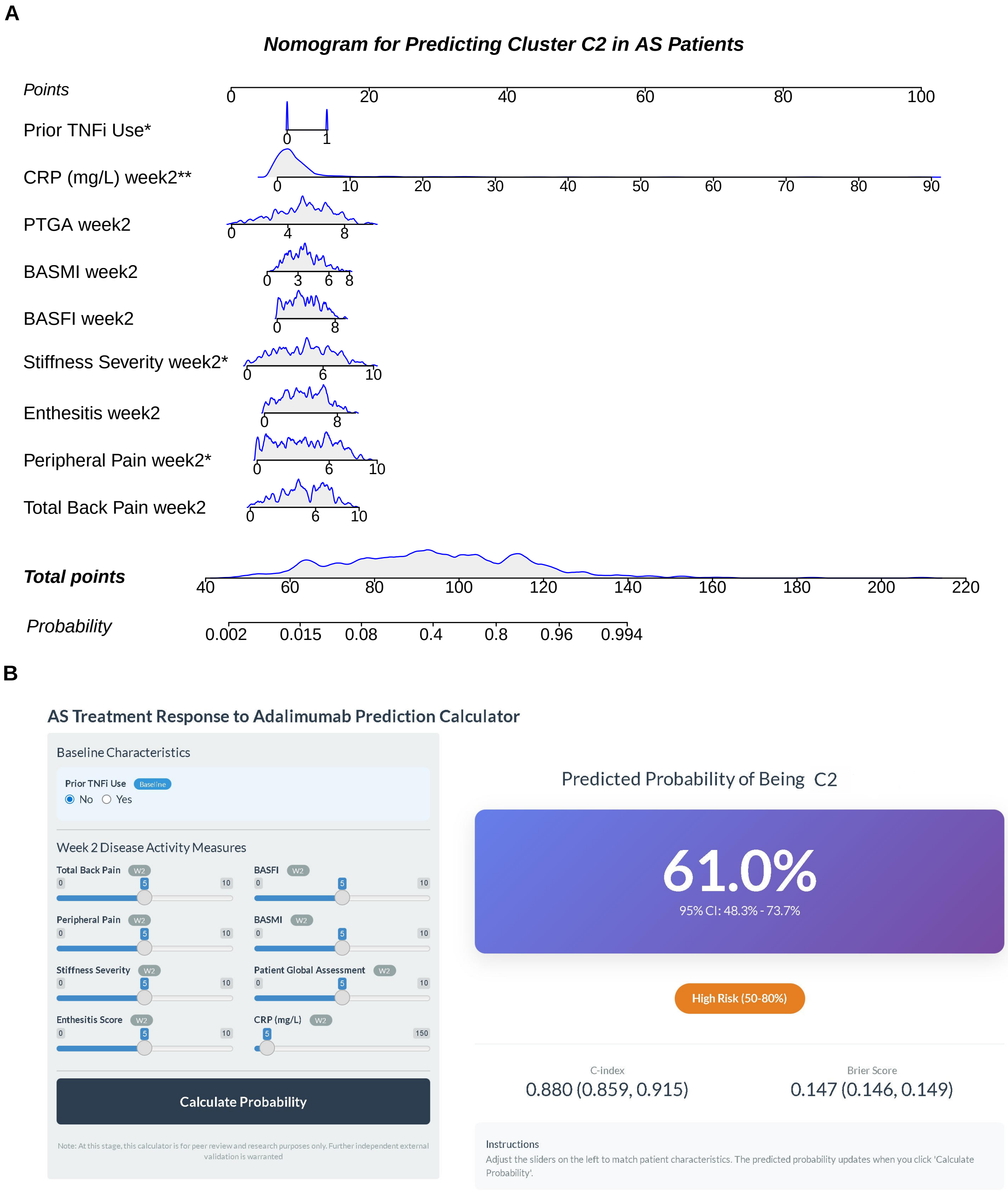

Supplementary Figure 16. Predictive Model of cluster C2 based on baseline and week 2 data, presented in Nomogram (A) and online shiny-based calculator (B)

References

1. Heijde D van der, Song I-H, Pangan AL, Deodhar A, van den Bosch F, Maksymowych WP, Kim T-H, Kishimoto M, Everding A, Sui Y, et al. Efficacy and safety of upadacitinib in patients with active ankylosing spondylitis (SELECT-AXIS 1): a multicentre, randomised, double-blind, placebo-controlled, phase 2/3 trial. *Lancet (Lond Engl)* (2019) 394:2108–2117. doi: 10.1016/S0140-6736(19)32534-6

2. Baeten D, Sieper J, Braun J, Baraliakos X, Dougados M, Emery P, Deodhar A, Porter B, Martin R, Andersson M, et al. Secukinumab, an Interleukin-17A Inhibitor, in Ankylosing Spondylitis. *N Engl J Med* (2015) 373:2534–2548. doi: 10.1056/NEJMoa1505066

3. Deodhar A, Désirée van der Heijde, Gensler LS, Kim T-H, Maksymowych WP, Østergaard M, Poddubnyy D, Marzo-Ortega H, Bessette L, Tomita T, et al. Ixekizumab for patients with non-radiographic axial spondyloarthritis (COAST-X): a randomised, placebo-controlled trial. *Lancet* (2020) 395:53–64. doi: 10.1016/S0140-6736(19)32971-X

4. Ratitch B, O'Kelly M, Tosiello R. Missing data in clinical trials: from clinical assumptions to statistical analysis using pattern mixture models. *Pharm Stat* (2013) 12:337–347. doi: 10.1002/pst.1549

5. Aleya Khalifa, Gloria HJ Graf. "Chapter 5 Methods.," *A Practical Guide to Sensitivity Analysis for Causal Effects in the Presence of Non-Ignorable Loss to Follow-Up* https://bookdown.org/glorya_hu/MNAR-Guide/methods.html

6. Buuren SV, Groothuis-Oudshoorn K. **mice** : Multivariate Imputation by Chained Equations in *R*. *J Stat Softw* (2011) 45: doi: 10.18637/jss.v045.i03

7. Dong Y, Peng C-YJ. Principled missing data methods for researchers. *SpringerPlus* (2013) 2:222. doi: 10.1186/2193-1801-2-222

8. Stef Van Buuren. "Model form and predictors.," *Flexible Imputation of Missing Data, Second Edition* https://stefvanbuuren.name/fimd/sec-modelform.html [Accessed April 9, 2026]

9. Rubin DB. *Multiple Imputation for Nonresponse in Surveys*. 1st ed. Wiley. (1987). doi: 10.1002/9780470316696

10. Monti S, Tamayo P, Mesirov J, Golub T. Consensus clustering: a resampling-based method for class discovery and visualization of gene expression microarray data. *Machine Learning* (2003) 52:91–118. doi: 10.1023/A:1023949509487

11. Kuhn M, Johnson K. *Applied predictive modeling*. New York, NY: Springer. (2013).

12. Steyerberg EW, Harrell FE, Borsboom GJ, Eijkemans MJ, Vergouwe Y, Habbema JD. Internal validation of predictive models: efficiency of some procedures for logistic regression analysis. *J Clin Epidemiol* (2001) 54:774–781. doi: 10.1016/s0895-4356(01)00341-9

13. Efron B, Tibshirani R. Improvements on Cross-Validation: The 632+ Bootstrap Method. *J Am Stat Assoc* (1997) 92:548–560. doi: 10.1080/01621459.1997.10474007

14. Stephen Rhodes. pminternal: Internal Validation of Clinical Prediction Models. (2025) doi: https://github.com/stephenrho/pminternal

15. Noma H, Shinozaki T, Iba K, Teramukai S, Furukawa TA. Confidence intervals of prediction accuracy measures for multivariable prediction models based on the bootstrap-based optimism correction methods. *Stat Med* (2021) 40:5691–5701. doi: 10.1002/sim.9148

16. Pencina MJ, D'Agostino RB, D'Agostino RB, Vasan RS. Evaluating the added predictive ability of a new marker: from area under the ROC curve to reclassification and beyond. *Stat Med* (2008) 27:157–172; discussion 207-212. doi: 10.1002/sim.2929
